# A Randomized, Crossover Study to Evaluate the Pharmacokinetics of Amantadine and Oseltamivir Administered Alone and in Combination

**DOI:** 10.1371/journal.pone.0001305

**Published:** 2007-12-12

**Authors:** Dennis Morrison, Sandip Roy, Craig Rayner, Ahmed Amer, Dan Howard, James R. Smith, Thomas G. Evans

**Affiliations:** 1 Bio-Kinetic Clinical Applications, Springfield, Missouri, United States of America; 2 Novartis Exploratory Clinical Development, Cambridge, Massachusetts, and East Hanover, New Jersey, United States of America; 3 F. Hoffmann-La Roche Ltd., Pharmaceuticals Division, Basel, Switzerland; Oxford University Clinical Research Unit, Viet Nam

## Abstract

The threat of potential pandemic influenza requires a reevaluation of licensed therapies for the prophylaxis or treatment of avian H5N1 infection that may adapt to man. Among the therapies considered for use in pandemic influenza is the co-administration of ion channel and neuraminidase inhibitors, both to potentially increase efficacy as well as to decrease the emergence of resistant isolates. To better understand the potential for drug interactions, a cross-over, randomized, open-label trial was conducted with amantadine, 100 mg po bid, and oseltamivir, 75 mg po bid, given alone or in combination for 5 days. Each subject (N = 17) served as their own control and was administered each drug alone or in combination, with appropriate wash-out. Co-administration with oseltamivir had no clinically significant effect on the pharmacokinetics (PK) of amantadine [mean ratios (90% CI) for AUC_0-12_ 0.93 (0.89, 0.98) and C_max_ 0.96 (0.90, 1.02)]. Similarly, amantadine co-administration did not affect oseltamivir PK [AUC_0-12_ 0.92 (0.86, 0.99) and C_max_ 0.85 (0.73, 0.99)] or the PK of the metabolite, oseltamivir carboxylate [AUC_0-12_ 0.98 (0.95, 1.02) and C_max_ 0.95 (0.89, 1.01)]. In this small trial there was no evidence of an increase in adverse events. Although many more subjects would need to be studied to rule out a synergistic increase in adverse events, the combination in this small human drug-drug interaction trial appears safe and without pharmacokinetic consequences.

**Trial Registration:**

ClinicalTrials.gov NCT00416962

## Introduction

The possible adaptation of avian influenza virus into an easily transmissible virus in humans, resulting in a potential pandemic of a magnitude not seen for nearly a century, has resulted in heightened awareness of influenza therapeutic options.[1] Only two classes of compounds are presently licensed for the treatment or prophylaxis of epidemic seasonal influenza, the ion channel inhibitors amantadine and rimantadine (active only against influenza A) and the neuraminidase inhibitors, oseltamivir and zanamavir.[2] Due to the increased resistance of circulating seasonal influenza strains against amantadine, the intrinsic resistance of most H5N1 strains, and the rapid emergence of resistance to ion channel inhibitors, amantadine is not considered to be a first line agent for use in pandemic influenza disease resulting from potential human adaptation followed by sustained human-to-human transmission of H5N1 avian influenza.[3], [4], [5] Because of its oral availability, activity against most strains of H5N1, and extensive safety data, oseltamivir, a prodrug of oseltamivir carboxylate, is considered the likely drug of choice for both treatment and prophylaxis for potential pandemic influenza at this time.[4]

However, as the range of available treatment options has broadened, the use of combination antiviral therapy has become standard of care in the management of some viral diseases, most notably RNA virus such as HIV. Combination antiretroviral therapy has been shown to be associated with greater and more prolonged reduction in viral burden and with reduced and delayed emergence of resistance. As some strains of H5N1 influenza virus remain sensitive to amantadine, the question has arisen as to whether the use of the oseltamivir and amantadine in combination may not represent an additional approach to improve efficacy outcomes and to prevent the emergence of resistance to either drug. Recent WHO guidelines for the pharmacological management of avian influenza virus infection in humans recommended that where the virus is sensitive to amantadine, that combination use of oseltamivir with amantadine could be considered. (WHO/PSM/PAR/2006.6)

These two classes of drugs have different pharmacological mechanisms of action and it has been speculated that a combination of the two agents might improve efficacy outcomes or increase the resistance barriers. In vitro and animal data has shown that there is some basis for both of these considerations.[6], [7], [8] An *in vitro* study of the combination revealed that the use of this combination in H5N1 drug-sensitive viruses prevented amino acid substitutions in the HA, NA or M proteins using relatively low concentrations of oseltamivir combined with amantadine, whereas each single agent resulted in some mutations of variable decreased susceptibility.[8] A second study by the same group showed that the combination of drugs *in vivo* was more effective at lowering viral load and preventing clinical illness and mortality in mice infected with H5N1 than when either oseltamivir or amantadine was used alone (Ilyushina NA et al, Amantadine-oseltamivir combination therapy for H5N1 influenza virus infection in mice. Antiviral therapy, In press.)

Other combinations of drugs used against influenza have been studied with similar results. Ribavirin and rimantadine have been shown to have a synergistic effect in vitro in MDCK cells, and ribavirin and amantadine were synergistic *in vivo* in mice.[7], [9] Rimantadine and zanamavir, or other combinations of M2 and neuraminidase inhibitors,[6] have been studied *in vitro* with promising results, and a small trial in humans was underpowered.[10]

Line listings obtained by the authors from post-marketing reports of the use of amantadine (Symmetrel, Novartis) or oseltamivir (Tamiflu, Roche) have shown that physicians, despite a lack of data or approval, have used these drugs in combination or series in treating seriously ill influenza patients.

For these reasons, it appeared prudent to produce data to evaluate the pharmacokinetics of the two drugs when used in combination, and to rule out an unexpected adverse event that may occur at a relatively high frequency. There are no theoretical considerations, using the known drug metabolism or excretion pathways of either drug (noting that oseltamivir is the prodrug of oseltamivir carboxylate), that lead to a prediction of meaningful pharmocological interactions. Nonetheless, the following trial was designed to provide some data in humans on the use of the drugs in combination, and to evaluate the pharmacokinetics of amantadine and oseltamivir administered alone and in combination.

## Methods

The protocol for this trial and supporting CONSORT checklist are available as supporting information; see Checklist S1 and Protocol S1.

### Participants

A total of 18 healthy subjects were to be enrolled, with replacement of drop-outs allowed. A single Phase 1 site was used. The study population was comprised of healthy males and female ages 18 to 45 years, and in good health as determined by past medical history, physical examination, vital signs, electrocardiogram, and laboratory tests. Female subjects of child bearing potential were required to use non-prescription protocol-indicated contraception, or be postmenopausal for at least 1 year prior to inclusion, as confirmed by a plasma follicle-stimulating hormone (FSH) concentration of >40 IU/L, or must have been surgically sterilized at least 6 months prior to screening. The body mass index (BMI) was required to be within the range of 18–30 kg/m^2^, with a minimum weight of 50 kg.

Subjects were excluded from entry into, or continuation in the study based on any of the following: if they were smokers (documented by urine cotinine levels for nicotine exposure); pregnant or lactating; using any prescription drugs in the 4 week period prior to dosing or used over-the-counter medication in the 2 week period prior to dosing; had participated in any clinical investigation in the 4 week period prior to dosing; had a significant illness within two weeks prior to dosing; had a past medical history of clinically significant ECG abnormalities, a family history of a prolonged QT-interval syndrome, autonomic dysfunction, acute or chronic bronchospastic disease, clinically significant drug allergy, atopic allergy; had any surgical or medical condition which may alter the absorption, distribution, metabolism or excretion of drugs; had evidence of liver disease or liver injury as indicated by abnormal liver function tests, impaired renal function, or an abnormal CBC. Exclusion criteria also existed for a history of immunodeficiency diseases, including a positive HIV, a positive Hepatitis B surface antigen (HBsAg) or Hepatitis C test, or a history of drug or alcohol abuse in the 12 months prior to dosing.

### Ethics

All subjects underwent informed consent approved by Bio-Kinetic Clinical Applications Institutional Review Board , an independent review Board.

### Interventions

Each subject participated in a screening period, three baseline periods, three 5-day treatment periods, two 5 to 7-day washout periods, and a study completion evaluation (Table 1). The duration of the treatment period was chosen to exceed the time required to reach a steady state concentration for amantadine, oseltamivir and oseltamivir carboxylate.

**Table 1:** Treatment sequences

Treatment: **Subjects randomized using the following scheme (with a wash-out period of 5 days between each treatment). In group B 3 subjects were randomized but only 2 entered.**				
Treatment Sequence	Sample Size	Period 1	Period 2	Period 3
A	3	Treatment 1	Treatment 2	Treatment 3
B	3 (2)	Treatment 1	Treatment 3	Treatment 2
C	3	Treatment 2	Treatment 1	Treatment 3
D	3	Treatment 2	Treatment 3	Treatment 1
E	3	Treatment 3	Treatment 1	Treatment 2
F	3	Treatment 3	Treatment 2	Treatment 1

Treatment 1 = Amantadine 100 mg BID for five days

Treatment 2 = Oseltamivir 75 mg BID for five days

Treatment 3 = [Amantadine 100 mg+Oseltamivir 75 mg] BID for five days

During recruitment and the baseline period, the subjects were informed to refrain from strenuous physical exercise for 7 days before dosing and until after the study completion, alcohol for 48 hours before dosing until after the study completion evaluation, or the intake of xanthine (e.g., caffeine) containing food or beverages 48 hours before dosing.

Subjects were randomized to one of six groups, each containing 3 participants (**Table 1**). During Period 1, subjects received the first dose of the assigned study drug(s) on Day 1 and remained domiciled for the full 5 days of each treatment period. Pre-dose pharmacokinetic samples were collected on the first day (Day 1) of each treatment. Pharmacokinetic assessments were performed as outlined below. The subjects returned to the study site for Treatment Periods 2 and 3 after a washout period of at least 5 days but no longer than 7 days.

On the evening of Day 4 of each treatment period, subjects fasted overnight prior to dosing and for 4 hours after drug administration. Meals were similar in caloric content for all subjects on the days of dosing. When mealtimes coincided with bleed times, blood was drawn before the meal was provided.

Study safety laboratories were performed at baseline of each treatment period and 12 hours after the completion of the last treatment period. These included: Hemoglobin, hematocrit, WBC count with differential as percentage and absolute value, RBC count and platelet count, albumin, alkaline phosphatase, total bilirubin, calcium, cholesterol, creatinine, CK, GGT, glucose, LDH, inorganic phosphorus, lipase, amylase, potassium, total protein, AST, ALT, sodium, triglycerides, urea and uric acid. Urinalysis was conducted for specific gravity, pH, semi-quantitative “dipstick” evaluation of glucose, protein, bilirubin, ketones, leukocytes, and blood. A microscopic examination including RBC, WBC, proteins, and casts was performed only when dipstick evaluation was positive for WBC, or proteins, or blood. An ECG was performed at baseline of each treatment period and 12 hours after the last treatment period completion.

### Pharmacokinetic evaluations and measurements

Blood collection occurred predose, 0.5, 1, 1.5, 2, 3, 4, 6, 8, 10 and 12 hr on the last day of each Treatment Period (Day 5), and pre-dose samples prior to morning and evening doses on the first day of each Treatment Period (Day 1). Plasma (∼1.5 mL) was separated by centrifugation within 60 minutes of blood collection, and the supernatant plasma carefully transferred into two separate 2-mL screw-cap, polypropylene vials. Plasma samples were immediately stored, in an upright position at or below −15°C for amantadine or −70°C for oseltamivir.

Amantadine and acetylamantadine plasma concentrations were determined using a validated liquid chromatography-tandem mass spectrometry (LC-MS/MS) method with an LLOQ of approximately 5.00 ng/mL for each analyte at the CEDRA Corporation in Austin, TX. Oseltamivir and oseltamivir carboxylate plasma concentrations were determined using a validated high–performance liquid chromatography/mass spectrometry method [11] with LLOQs of approximately 1 ng/mL and 10 ng/mL, respectively, at Bioanalytical Systems Ltd. in the UK. Performance data are as given in [12].

### Objectives

The study was carried out with the primary endpoint to characterize the pharmacokinetics in healthy adult volunteers of amantadine (100 mg BID) and oseltamivir (75 mg BID) when administered alone or in combination. A secondary objective was to assess the safety and tolerability of twice daily oseltamivir and amantadine when given alone and when given in combination; however, the trial was not powered for adverse event endpoints. The trial was an open-label, multiple dose, randomized, three-way crossover study.

### Randomization

Randomization was performed by Novartis Drug Supply Management using a validated system in which each subject was assigned to one of the 6 study arms. The trial was open label, and thus no blinding was needed.

### Statistical methods

To compare the results of the individual versus combination therapy arms for both amantadine and oseltamivir, log-transformed PK parameters AUC and Cmax were analyzed by a linear mixed effect model, with fixed effects from sequence, treatment, and period, and random effects from subject nested in sequence. Estimators for the treatment difference, including the corresponding 90% confidence intervals, were obtained based upon the log-transformed observations. The estimators and confidence intervals were then “back transformed” to the original scale. The resulting 90% confidence interval of the appropriate treatment mean ratios were used to explore the drug-drug interactions.

## Results

### Demographics and study conduct

Recruitment occurred in the Fall of 2006. Only 17 of the 18 subjects were enrolled (out of 27 screened), as one subject dropped out before the first dose (in arm B, Table 1). There were 4 women and 13 men, with a mean age of 27.9 (range 22–41). All subjects were able to go through the entire dosing cohort, with no drop-outs during the trial. No subject dropped out due to an adverse event or intolerance of the medications.

#### Effect of oseltamivir on amantadine pharmacokinetics

**Figure 1:**
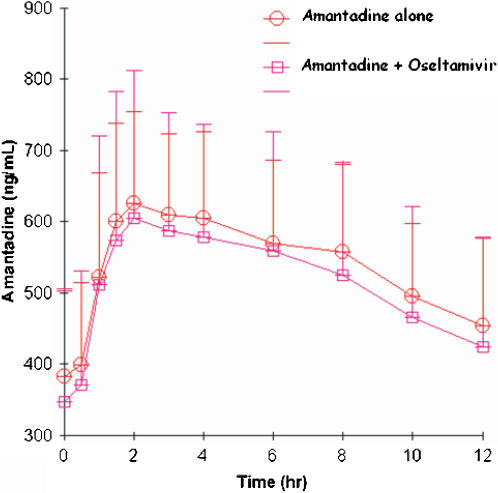
Plasma amantadine concentrations (mean values±standard deviation) following administration of 100-mg BID amantadine for five days (open circles) or 100 mg BID amantadine plus 75 mg BID oseltamivir (open squares) in 17 healthy volunteers

Amantadine concentration-time profiles after administration of 100 mg BID for five days and during coadministration with 75 mg oseltamivir BID dosing for five days are shown in Figure 1. Amantadine AUC_0-12_ and C_max_ decreased slightly when coadministered with oseltamivir compared to amantadine administration alone (Table 2). The geometric mean ratio (GMR) of the AUC_0-12_ of amantadine, coadministered with oseltamivir compared to amantadine alone, was 0.93. GMR of the C_max_ and C_trough_ were 0.96 and 0.92 , respectively. As expected for amantadine, the geometric mean values for apparent clearance (15.6 vs 16.7 L/hr), apparent volume of distribution (332 vs 326 L), half-life (14.7 vs 13.6 hr) and Tmax (2.1 vs 2.4 hr) were also similar between amantadine monotherapy versus combination therapy with oseltamivir.

**Table 2:** Pharmacokinetics of amantadine after 100-mg BID for five days or when coadministered with 75-mg oseltamivir BID for five days (N = 17)

PK Parameters for Amantadine	N		Geometric Means		Ratio of Geometric Means	90% Confidence Intervals
	Amantadine alone	Amantadine + Oseltamivir	Amantadine alone	Amantadine + Oseltamivir	Amantadine + Oseltamivir/Amantadine alone	
Cmax (ng/mL)	17	17	636.2	611.3	0.96	0.90, 1.02
AUCtau (ng.hr/mL)	17	17	6413.6	5992.8	0.93	0.89, 0.98
Clast (ng.hr/mL)	17	17	438.1	401.6	0.92	0.86, 0.98

#### Effect of amantadine on oseltamivir pharmacokinetics

**Figure 2:**
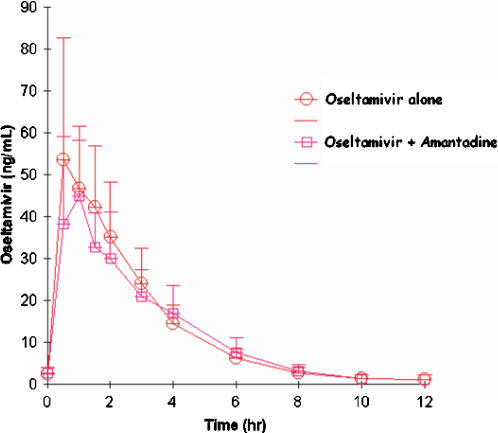
Plasma oseltamivir concentrations (mean values±standard deviation) following administration of 100-mg BID amantadine for five days (open circles) or 100 mg BID amantadine plus 75 mg BID oseltamivir (open squares) in 17 healthy volunteers

**Figure 3:**
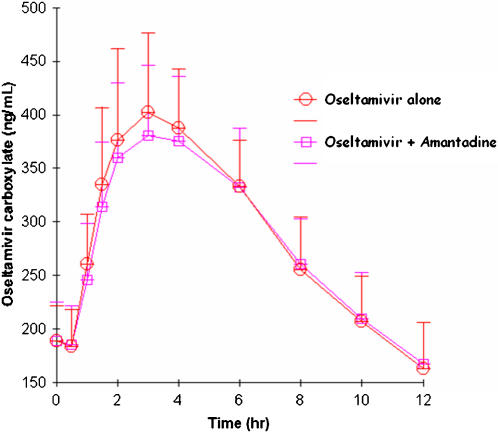
Plasma oseltamivir carboxylate concentrations (mean values±standard deviation) following administration of 75-mg BID oseltamivir for five days (open circles) or 75 mg BID oseltamivir plus 100 mg BID amantadine (open squares) in 17 healthy volunteers

Oseltamivir and oseltamivir caroboxylate concentration-time profiles after administration of 75 mg BID for five days and during coadministration with 100 mg amantadine BID dosing for five days are shown in Figures 2 and 3, respectively. Oseltamivir AUC_0-12_ and C_max_ decreased slightly when coadministered with amantadine compared to oseltamivir administration alone (Table 3 and 4). The geometric mean ratio (GMR) of the AUC_0-12_ of oseltamivir, coadministered with oseltamivir compared to amantadine alone, was 0.92. GMR of the C_trough_ and C_max_ were 0.95 (with a lower confidence interval of .73) and 0.85 (with a lower confidence interval of 0.79), respectively. The AUC_0-12_ and C_max_ of the active oseltamivir carboxylate also decreased slightly when coadministered with amantadine compared to oseltamivir administration alone (Table 4). The geometric mean ratio (GMR) (90% confidence interval [CI]) of the AUC_0-12_ of oseltamivir carboxylate, coadministered with oseltamivir compared to amantadine alone, was 0.98. GMR of the C_max_ and C_trough_ were 0.95 and 1.03, respectively. As expected, the geometric mean values for oseltamivir apparent clearance (465 vs 505 L/hr), apparent distribution volume (1245 vs 1329 L), half-life (1.85 vs 1.83 hr) and Tmax (0.76 vs 0.85 hr) were also similar between oseltamivir monotherapy versus combination therapy with amantadine. In addition, geometric mean values for oseltamivir carboxylate apparent clearance (21.9 vs 22.3 L/hr), apparent distribution volume (192 vs 197 L), half-life (6.09 vs 6.15 hr) and Tmax (3.22 vs 3.07 hr) were similar between oseltamivir monotherapy versus combination therapy with amantadine.

**Table 3:** Pharmacokinetics of oseltamivir following 75-mg BID for five days administered alone or when coadministered with 100-mg amantadine BID for five days (N = 17)

PK Parameters for Oseltamivir	N		Geometric Means		Ratio of Geometric Means	90% Confidence Intervals
	Oseltamivir alone	Amantadine + Oseltamivir	Oseltamivir alone	Amantadine + Oseltamivir	Amantadine + Oseltamivir/Oseltamivir alone	
Cmax (ng/mL)	17	17	60.2	51.1	0.85	0.73, 0.99
AUCtau (ng.hr/mL)	17	17	161.3	148.6	0.92	0.86, 0.99
Clast (ng.hr/mL)	17	17	1.7	1.6	0.95	0.79, 1.14

**Table 4:** Pharmacokinetics of oseltamivir carboxylate following administration of oseltamivir 75-mg BID for five days alone or when coadministered with 100-mg amantadine BID for five days (N = 17)

PK Parameters for Oseltamivir carboxylate	N		Geometric Means		Ratio of Geometric Means	90% Confidence Intervals
	Oseltamivir alone	Amantadine + Oseltamivir	Amantadine alone	Amantadine + Oseltamivir	Amantadine + Oseltamivir/Oseltamivir alone	
Cmax (ng/mL)	17	17	407.1	385.6	0.95	0.89, 1.01
AUCtau (ng.hr/mL)	17	17	3429.1	3369.3	0.98	0.95, 1.02
Clast (ng.hr/mL)	17	17	157.8	162.8	1.03	0.99, 1.08

### Safety

Only eight adverse events, all mild, were reported during the trial, two with amantadine alone, and three with oseltamivir alone or in combination. One episode of mild upset stomach was associated with the combination, was mild, and resolved without treatment.

## Discussion

Many countries worldwide have stockpiled both amantadine and oseltamivir for use as either for prophylaxis or treatment of potential pandemic influenza. Guidelines have also been established by WHO for the treatment of persons infected with avian H5N1 influenza A virus, despite a dearth of information on the clinical effectiveness of the proposed recommendations. Included in these guidelines is the statement that “clinicians might administer a combination of a neuraminidase inhibitor and an M2 inhibitor if local surveillance data show that the H5N1 is known or likely to be susceptible”. There are, however, no data to support whether there may be an unanticipated drug-drug-interaction which might impact on the posology of such a combination.

Excretory and metabolic processes involved in clearance of amantadine and oseltamivir are different. Amantadine is primarily excreted unchanged in the urine by glomerular filtration and tubular secretion, whereas oseltamivir is extensively converted to oseltamivir carboxylate by esterases.[13], [14] Neither oseltamivir nor oseltamivir carboxylate is a substrate for, or inhibitor of, cytochrome P450 isoforms.

As expected, coadministration with oseltamivir had no meaningful impact on the pharmacokinetics of amantadine. Similarly, amantadine coadministration did not meaningfully affect oseltamivir PK or the PK of metabolite, oseltamivir carboxylate. Except for minor changes in oseltamivir C_max_ and C_trough_, confidence intervals for geometric mean ratios of all other parameters were within bioequivalence criteria of 80–125%, and the difference observed for oseltamivir were not considered clinically meaningful as the point estimates were near unity and the study size was relatively small.

In a study conducted with amantadine, plasma acetylamantadine accounted for up to 80% of the concurrent amantadine plasma concentration in 5 of 12 healthy volunteers. Acetylamantadine was not detected in the plasma of the remaining seven volunteers (see Symmetrel® package insert) In the current study, acetylamantadine was not detected in the plasma of any of the seventeen volunteers that participated in this study. In another study, after 15 days of amantadine 100 mg b.i.d., the C_max_ was 0.47±0.11 µg/mL in four of the five volunteers (Symmetrel® package insert). In this study we report a higher geometric mean of C_max_ of 0.64 µg/mL after 5 days of amantadine 100 mg b.i.d and a geometric mean C_max_ of 0.61 µg/mL when coadministered with oseltamivir.

Previously reported mean C_max_ of 65.2 ng/mL was observed for oseltamivir and 348 ng/mL for oseltamivir carboxylate after twice daily oral dosing with 75 mg oseltamivir capsule (Oseltamivir package insert). In this study we report a geometric mean C_max_ of 60.2 ng/mL for oseltamivir and 407.1 ng/mL for oseltamivir carboxylate following administration of 75 mg BID for five day. When co-administered with amantadine we observed geometric mean C_max_ of 51.1 ng/mL for oseltamivir and 385.6 ng/mL for oseltamivir carboxylate. Mean AUC_0-12_ values observed in this study (geometric mean of 161.3 ng.hr/mL for oseltamivir and 3429.1 ng.hr/mL for oseltamivir carboxylate) were also slightly higher than values reported earlier for oseltamivir (112 ng.hr/mL) and oseltamivir carboxylate (2719 ng.hr/mL). When co-administered with amantadine we report geometric mean AUC_0-12_ of 148.6 ng.hr/mL for oseltamivir and 3369.3 ng.hr/mL for oseltamivir carboxylate.

The study did not have sufficient power to examine the potential increase in adverse events, especially those related to the CNS. Amantadine is well known to result in alteration of mental status, especially in the elderly, but that population was not studied here. The potential for a CNS interaction with neuraminidase inhibitors remains unproven, although a new precaution has recently been added to the US label of oseltamivir (http://www.fda.gov/ohrms/dockets/ac/06/briefing/2006-4254B_09_05-PI-clean-110906.pdf).

Most H5N1 isolates have been resistant to amantadine. Recently, the H5N1 isolates have been grouped into two distinct clades and 3 subclades sub-lineages. These include the clade 1 strains that are insensitive to amantadine and the clade 2 viruses circulating in Indonesia which show an approximate 50% resistance to amantadine.[15] It is not known if amantadine resistant viruses will persist or be replaced by fully susceptible strains (WHO report 2006). Recently, a subclade has been reported from China (Fujan), but the amantadine sensitivity of this strain is not yet clear.

Oseltamivir is effective against all strains of influenza A and B tested, including H5N1.

The neuraminidase of H5N1 appears to be have increased in sensitivity over time to NA inhibitors since these viruses emerged in 1997.[4] Seasonal influenza strains have a low rate of resistance to oseltamivir-0.33% in adults and 4% in children, although some studies have reported higher rates in children who may have been underdosed.[16] In patients with H5N1 infection viral load appears to be much higher than encountered in seasonal influenza, and a total of 3 cases have been reported where reduced susceptibility to oseltamivir was noted.[17], [18] These results highlight not only the need to understand and administer the correct oseltamivir dosage as early as possible but also the need for continued vigilance in H5N1 infected patients and the need for additional antiviral agents or combinations.

Combination treatment in mice using equivalent human therapeutic doses of oseltamivir and amantadine against a lethal challenge of recombinant amantadine sensitive A/Vietnam/1203/04 (H5N1) virus protected 90% of the animals in contrast to dosing of each as a single agent where the mortality protection level was only 20% (Ilyushina, in press). No benefit of the combination was observed when infection was performed with an amantadine-resistant virus. The pharmacological basis for this beneficial combination of the two therapeutics might be explained by their targeting different viral proteins (NA and M2) , but this is not clear at this time.

In summary, the combination of amantadine and oseltamivir was without evidence of an increase in adverse events (although underpowered for this endpoint), and there was no clinically significant effect of either drug on the pharmacokinetic profile of the other. Data from this limited human volunteer study and the preliminary animal and in vitro data available on amantadine and oseltamivir indicate that it would be worthwhile to conduct further studies using such a combination.

## Supporting Information

Protocol S1 Trial protocol
(0.17 MB PDF)

Checklist S1 CONSORT Checklist
(0.05 MB DOC)
