# Supplementary material for: A Randomized, Crossover Study to Evaluate the Pharmacokinetics of Amantadine and Oseltamivir Administered Alone and in Combination
Source: PLoS One. 2007 Dec 12;2(12):e1305. doi: 10.1371/journal.pone.0001305 (PMC2110886; doi:10.1371/journal.pone.0001305)
Supplement: Protocol S1 — Trial protocol (0.17 MB PDF) [file pone.0001305.s001.pdf]

NOVARTIS

Exploratory Clinical Development

SYO380

Protocol No. CSYO380A2101

An open-label, multiple dose, randomized, three-period crossover study in healthy volunteers to evaluate the effect of co-administration of amantadine 100 mg BID and oseltamivir 75 mg BID on the pharmacokinetic properties of amantadine and oseltamivir.

Author(s): Roy S, Howard D, Evans T, Amer A, Dole K, Wang M

Document type: Clinical study protocol

Development Phase: Phase I

Release date: 04-Aug-2006

Property of Novartis
It may not be used, divulged, published or otherwise disclosed
without the consent of Novartis

Notification of serious adverse events

A serious adverse event (SAE) is any event which is fatal or life-threatening, which requires or prolongs hospitalization, which is significantly or permanently disabling or incapacitating, which constitutes a congenital anomaly or a birth defect, or which is medically significant, may jeopardize the subject and may require medical or surgical intervention to prevent one of the outcomes listed above.

Any SAE occurring in a subject after first dosing with study drug and until 30 days after stopping the trial must be reported.

The investigator will ensure that the **SAE reporting form** is completed and **faxed** by the **investigator to the local Novartis Clinical Safety and Epidemiology (CS&E) Department within 24 hours** of learning of the occurrence of any SAE even if the SAE does not appear to be drug-related**.** The original form of the Serious Adverse Event Form, together with the fax confirmation sheet, must be kept with the case report forms at the study site.

More details in **Sections 8 and 9** of this protocol.

Fax numbers of local Novartis Clinical Safety & Epidemiology Departments

**North America**	**Fax (++ 1) 973 781 5775 or**<br>**(++ 1) 888 299 4565 (toll free number)**

In addition, the investigator will inform one of the following responsible persons of Novartis Exploratory Clinical Development:

**Ahmed Amer**	**Tel. Business: +1 862 778 1333**
**Exploratory Clinical Development**	**Fax. Business: +1 973 781 2077**
**Clinical Study Leader**	

**Thomas Evans**	**Tel. Business: +1 617 871 7698**
**Exploratory Clinical Development**	**Fax. Business: +1 617 871 7629**
**Clinical Pharmacology Expert**	**Tel. Mobile phone: +1 617 230 6589**

Table of contents

Table of contents 3
List of tables 5
List of abbreviations 5
Pharmacokinetic definitions and symbols 7
Glossary of terms 8
Study synopsis and assessment schedule 10
1 Background 15
1.1 Introduction 15
1.2 Summary of relevant data 16
1.3 Study and dose rationale 17
2 Study purpose 17
3 Objectives 17
3.1 Primary objectives 17
3.2 Secondary objective 17
4 Overview of study design 18
5 Population 19
5.1 Entry criteria 19
5.1.1 Inclusion criteria 20
5.1.2 Exclusion criteria 20
5.2 Dietary, fluid and other restrictions 22
6 Treatment 22
6.1 Study drugs 22
6.2 Treatment assignment 23
6.3 Treatment blinding 23
6.4 Emergency unblinding of treatment assignment 23
6.5 Treating the subjects 23
6.5.1 Study drug administration 23
6.5.2 Permitted study drug adjustments 24
6.5.3 Concomitant treatment 24
6.5.4 Recommended treatment of adverse events 24
6.5.5 Study drug discontinuation 24
6.5.6 Premature subject withdrawal 24
7 Assessments 25
7.1 Background, demographic and administrative assessments 25

7.2 Safety and tolerability assessments 27
7.3 Efficacy assessments 29
7.4 Pharmacokinetics 29
7.5 PK sample handling, labeling and shipment instructions 30
7.5.1 Sample labeling 30
7.5.2 Shipment of pharmacokinetic samples 30
7.5.3 Analytical method(s) 32
7.6 Pharmacodynamic assessments 33
7.7 Biomarkers 33
7.8 Tolerability 33
8 Safety monitoring 33
8.1 Reporting of adverse events 33
8.1.1 Definitions 33
8.1.2 Instructions for completing adverse event case report forms 34
8.2 Recommended treatment for known AEs 36
8.3 Data Monitoring Board 36
9 Serious adverse events reporting 36
9.1 Reporting procedures for SAEs 37
9.2 Pregnancies 37
10 Data review and database management 37
10.1 Site monitoring 37
10.2 Data collection and retention of documents 38
10.3 Auditing procedures 39
10.4 Database management and quality control 39
11 Data analysis 39
11.1 Populations for analysis 39
11.2 Sample size determination 40
11.3 Power for analysis of critical secondary variables 40
11.4 Subjects demographics/other baseline characteristics 40
11.5 Safety and tolerability data analysis 40
11.5.1 Safety and tolerability variables 40
11.6 Pharmacokinetic data analysis 41
11.7 Pharmacodynamic data analysis 42
11.8 Pharmacokinetic-pharmacodynamic data analysis 42
11.9 Biomarkers analysis 42
11.10 Health-related Quality of Life 42

11.11 Interim analysis 42
12 References (Available upon request) 43
13 Responsible study personnel....44
14 Appendix 1: Administrative procedures 46
14.1 Regulatory and ethical compliance....46
14.2 Responsibilities of the investigator and IRB/IEC/REB 46
14.3 Informed consent 46
14.4 Informed consent procedures....47
14.5 Amendments to the protocol....47
14.6 Administrative Amendment 47
14.7 Discontinuation of the study....48
14.8 Publication of results 48
15 Appendix 2: Treatment assignment 49
16 Appendix 3: Blood log....50
17 Appendix 4: PK samples shipping log....51
18 Appendix 5 Determination of body weight by height criteria 53
18.1 Determination of Body Mass Index (weight[kg] / height[m]^2) 53

List of tables

Table 4-1 Treatment sequences 18
Table 4-2 Treatment period overview 19

List of abbreviations

AE	adverse event
ALT	alanine aminotransferase
AP	alkaline phosphatase
AST	aspartate aminotransferase
AUC	area under the concentration time curve
BA	bioavailability
BE	bioequivalence
b.i.d.	bis in die / twice a day
bpm	beats per minute
BUN	blood urea nitrogen

CK	creatine kinase
CTL	clinical trial leader
CRF	Case Report / Record Form: It is used for either paper and electronic forms (eCRFs) unless specified otherwise
CRO	Contract Research Organization
CS&E	Clinical Safety and Epidemiology
ECD	Exploratory Clinical Development
ECG	electrocardiogram
EDC	Electronic Data Capture
FDA	Food and Drug Administration
GCP	good clinical practice
GGT / γ-GT	gamma-glutamyl-transpeptidase
HBsAg	hepatitis B surface antigen
HCV	hepatitis C virus
HIV	human immunodeficiency virus
IEC	Independent Ethics Committee
IND	Investigational New Drug application
IRB	Institutional Review Board
i.v.	intravenous(ly)
LDH	lactate dehydrogenase
LLLOQ	lower limit of quantification
mm Hg	millimeters of mercury
NCS	not clinically significant
NOAEL	no-observable adverse effect level
NTEL	no-toxic-effect level
o.d.	once a day
PD	pharmacodynamics
q.i.d.	quarter in die / four times a day
p.o.	oral
RBC	red blood cells (erythrocytes)
SAE	serious adverse event

SOP	Standard Operating Procedure
TBD	to be determined
t.i.d.	ter in die / three times a day
WBC	white blood cells (leukocytes)
WHO	World Health Organization

Pharmacokinetic definitions and symbols

Ae_{0-t}	amount of drug excreted into the urine from time zero to time 't' where t is the last sampling time point [mass units]
$Ae_{0-\infty}$	amount of drug excreted into the urine from time zero to infinity [mass units]
$AUC_{0-\infty}$	AUC from time zero to infinity [mass x time x volume^{-1}]
AUC_{τ}	AUC during a dosing interval (τ) at steady-state [amount x time x volume^{1}]
C_{max}	maximum (peak) observed plasma, blood, serum, or other body fluid drug concentration after single dose administration [mass x volume^{-1}]
C_{ss}	steady-state drug concentration in the plasma, blood, serum, or other body fluid during constant rate infusion [amount x volume^{-1}]
$C_{avg, ss}$	average steady-state drug concentration in the plasma, blood, serum, or other body fluid during multiple dosing [amount x volume^{-1}]. This was estimated as AUC_{τ} / τ
$C_{max, ss}$	maximum (peak) observed steady-state drug concentration in the plasma, blood, serum, or other body fluid during multiple dosing [amount x volume^{-1}]
$C_{min, ss}$	minimum (trough) observed steady-state drug concentration in the plasma, blood, serum, or other body fluid during multiple dosing [amount x volume^{-1}]
CL	total body clearance of drug from the plasma [volume x time^{-1}]<br>*Note*: clearance values from other body fluids may be noted by use of subscripts, e.g. CL_b refers to clearance from the blood and CL_u clearance of unbound drug from the plasma. If the clearance is following an extravascular dose and bioavailability is not known, the notation should be CL/F.
CL_R	renal clearance of drug [volume x time^{-1}] .
Fluctuation Index	calculated from steady-state concentrations (multiple dose) $[C_{max}-C_{min}]/A_{verage}$
k	first-order elimination rate constant [time^{-1}]

k_a	absorption rate constant (first-order) [time^{-1}]
k_e	rate constant (first-order) for the appearance of unchanged drug in the urine [time^{-1}] (Renal excretion rate constant)
λ_z	smallest (slowest) disposition (hybrid) rate constant [time^{-1}] may also be used for terminal elimination rate constant [time^{-1}]
MRT	mean residence time
R	accumulation ratio
t	time after drug administration [time]
t_{lag}	lag-time [time]
t_{max}	time to reach maximum (peak) plasma, blood, serum, or other body fluid drug concentration after single dose administration [time]
$t_{max,ss}$	time to reach maximum (peak) steady-state concentration in plasma, blood, serum, or other body fluid during multiple dosing [time]
$t_{½}$	the elimination half-life associated with the terminal slope (λ_z) of a semilogarithmic concentration-time curve [time]. Use qualifier for other half-lives.
τ	dosing interval [time]
V_{ss}	the apparent volume of distribution at steady-state [volume]

Glossary of terms

Control; control drug	A study drug used as a comparator to reduce assessment bias, preserve blinding of investigational drug, assess internal study validity, and/or evaluate comparative effects of the investigational drug
Screening	Point/time of subject/patient entry into the study; the point at which informed consent must be obtained (i.e., prior to starting any of the procedures described in the protocol)
Investigational drug	The study drug whose properties are being tested in the study; this definition is consistent with US CFR 21 Section 312.3 and is synonymous with “investigational new drug”.
Phase	A major subdivision of the study timeline; begins and ends with major study milestones such as, screening, or study completion.
Treatment Period	A minor subdivision of the study timeline that divides phases into smaller functional segments, i.e., the time starting from Baseline prior first study drug administration until the last day prior the next Baseline, or the Study completion visit.

Premature subject/patient withdrawal	Point/time when the subject/patient exits from the study prior to the planned completion of all study drug administration and assessments. At this time all study drug administration is discontinued. Study completion assessments must be completed.
Randomization number	A unique identifier assigned to each randomized subject/patient, corresponding to a specific treatment group assignment.
Study drug	Any drug administered to the subject/patient as part of the required study procedures; includes investigational drug and any control and comparator drugs.
Study drug discontinuation	Point/time when subject/patient permanently stops taking study drug for any reason; may or may not also be the point/time of premature subject withdrawal

Study synopsis and assessment schedule

SYO380

Title of study: An open-label, multiple dose, randomized, three-period crossover study in healthy volunteers to evaluate the effect of co-administration of amantadine 100 mg BID and oseltamivir 75 mg BID on the pharmacokinetic properties of amantadine and oseltamivir.

Planned dates: first subject dosed: 09-Sep-2006 last subject completed: 03-Oct-2006

Objectives:

Primary objectives

- To characterize the pharmacokinetics of amantadine 100 mg following twice daily administration alone or in combination with twice daily oseltamivir 75 mg in healthy volunteers.
- To characterize the pharmacokinetics of oseltamivir 75 mg following twice daily administration alone or in combination with twice daily amantadine 100 mg in healthy volunteers.

Secondary objective

- To assess the safety and tolerability of twice daily oseltamivir 75 mg and twice daily amantadine 100 mg when given alone and when given in combination.

Design: The study employs an open-label, multiple dose, randomized, three-period crossover design. A total of 18 healthy male or female volunteers will be enrolled. Each subject will participate in a screening period, three baseline periods, three 5-day treatment periods, two 5 to 7-day washout periods, and a study completion evaluation.

The following treatments will be administered during the study: amantadine or oseltamivir or both.

Treatment Sequence	Sample Size	Period 1	Period 2	Period 3
A	3	Treatment 1	Treatment 2	Treatment 3
B	3	Treatment 1	Treatment 3	Treatment 2
C	3	Treatment 2	Treatment 1	Treatment 3
D	3	Treatment 2	Treatment 3	Treatment 1
E	3	Treatment 3	Treatment 1	Treatment 2
F	3	Treatment 3	Treatment 2	Treatment 1

Treatment 1 = Amantadine 100 mg BID for five days
Treatment 2 = Oseltamivir 75 mg BID for five days
Treatment 3 = [Amantadine 100 mg + Oseltamivir 75 mg] BID for five days

Screening for this study will occur within 21 days of the first dose. Subjects will be admitted to the study site on Day -1 for baseline safety assessments and to confirm eligibility. Subjects who meet the inclusion/exclusion criteria at the first baseline will be randomized to one of the six sequences above.

During Period 1, subjects will receive the first dose of the assigned study drug(s) on Day 1 and remain domiciled for the full 5 days of each treatment period. Dosing will occur on each day between 0800 and 0830 for the morning dose(s) and between 2000 and 2030 for the evening dose(s). Pre-dose pharmacokinetic samples will be collected on the first day of each Treatment Period. Safety assessments will be performed during the 5-day domiciled treatment period. On the evening of Day 4, subjects will fast overnight (10 to 12 hours) prior to dosing and for 4 hours after drug administration. Pharmacokinetic assessments will start pre-dose and continue up to 12 hours post dose on Day 5.

Subjects will be discharged from the study center after the last pharmacokinetic and safety assessments have been performed. Subjects will return to the study site for Treatment Periods 2 and 3 after a washout period of at least 5 days but no longer than 7 days.

Serial pharmacokinetic blood sampling will occur on the last day of each Treatment Period up to 12 hours following drug administration. In addition, pre-dose samples will be collected prior to the morning and evening doses on the first day of each Treatment Period. On the serial pharmacokinetic sampling day, subjects will only receive the morning dose of study drug(s).

Subjects will be discharged from the study center after the last pharmacokinetic and safety assessments, which will include physical examinations, ECG, vital signs, clinical laboratory evaluations and adverse events monitoring.

Subjects who wish to discontinue the study or, who are withdrawn from the study by the investigator for any reason, will be asked to complete the End of Study assessments and may be replaced.

Treatment Period overview: the 3 Treatment Periods will follow this schedule

Day -1	Day 1 to 5	
Baseline	Treatment 1, 2 or 3	5-7 Days Washout Period
	Predose PK on Day 1*<br>Serial PK sampling on Day 5*	

*exact times are indicated in the assessment schedule

Planned number of subjects:

18 subjects will be enrolled in this study.

Inclusion criteria:

Healthy male or female subjects aged 18-45, inclusive, with a BMI of 18-30 kg/m^2 and who have provided written informed consent for participation in the study. In addition, all subjects must be in good health based on medical history, physical examination, vital signs, electrocardiogram and clinical laboratory tests.

**Investigational drug:**			
Amantadine	Tablet	100 mg	Oral
**Comparator drug:**			
Oseltamivir	Capsule	75 mg	Oral

Duration of treatment: 3 five-day Treatment Periods separated by 2 five to seven-day washout periods

Five days of treatment with oseltamivir: 75 mg b.i.d.

Five days of treatment with amantadine: 100 mg b.i.d.

Five days of treatment with oseltamivir 75 mg b.i.d. + amantadine 100 mg b.i.d.

Assessments and evaluations:

Background, demographic and administrative assessments

- Inclusion/exclusion criteria; Relevant medical history/Current medical conditions: Screening, review at Baseline prior to Treatment Period 1.
- Demography: Screening.
- Physical examination: Screening, Baseline of Treatment Period 1, Study Completion.
- Hepatitis screen, HIV screen: Screening.
- Alcohol test, Drug screen, Urine cotinine: Screening, Baseline of all Treatment Periods.
- Pregnancy test: Screening, Baseline of all Treatment Periods, Study Completion.
- Meal record: Days 5 (the last day of each Treatment Period).
- Drug administration record: each time study drug is administered.
- Study Completion information: Study Completion.
- Comments: as required.

Safety and tolerability assessments

- Vital signs and body measurements
    - Body height: Screening.
    - Body weight: Screening, Baseline prior to Treatment Period 1, and, Study Completion.
    - Body temperature: Screening, Baseline prior to Treatment Period 1, and, Study Completion.
    - Blood pressure, pulse rate: Screening*, Baseline prior to all Treatment Periods*, predose and 2 hours postdose on the first and last day (Day 1 and Day 5) of each Treatment Period; and Study Completion.

  (Note: measurements are supine, or supine and after 3 min standing if indicated by an *asterisk)
- ECG evaluation: Screening, Baseline prior to all Treatment Periods, and Study Completion.
- Hematology; Blood chemistry; Urinalysis: Screening, Baseline prior to all Treatment Periods, and Study Completion.
- Adverse events: from time of first administration of study drug until Study Completion. Adverse events occurring before starting study treatment but after signing the informed consent form are recorded on the Medical History/Current Medical Conditions Case Report Form.
- Serious adverse events: from time of first dosing until 4 weeks after Study Completion.
- Concomitant medications/Significant non-drug therapies: from time of first administration of study drug until Study Completion.
- Medication taken prior to first dosing: All prescription medications taken within one month, and over-the-counter drugs (including vitamins) taken within 14 days prior to dosing and throughout the study must be recorded on the Concomitant Medications/ Non-Drug Therapies page of the CRF.

Pharmacokinetic assessments

Blood Collections

Blood collection for amantadine: (3 mL plasma, EDTA plastic tubes): At predose, 0.5, 1, 1.5, 2, 3, 4, 6, 8, 10 and 12 hr on the last day of each Treatment Period (Day 5), and pre-dose samples prior to morning and evening doses on the first day of each Treatment Period (Day 1).

Blood collection for oseltamivir: (3 mL plasma, EDTA plastic tubes): At predose, 0.5, 1, 1.5, 2, 3, 4, 6, 8, 10 and 12 hr on the last day of each Treatment Period (Day 5), and pre-dose samples prior to morning and evening doses on the first day of each Treatment Period (Day 1).

Analytes

Amantadine media and methods: amantadine and acetylamantadine plasma concentrations will be determined using a validated liquid chromatography-tandem mass spectrometry (LC-MS/MS) method with an LLOQ of approximately 5.00 ng/mL for each analyte.

Oseltamivir media and methods: oseltamivir and oseltamivir carboxylate plasma concentrations will be determined using a validated high–performance liquid chromatography/mass spectrometry method with LLOQs of approximately 1 ng/mL and 10 ng/mL respectively.

Primary PK Parameters:

Steady state PK parameters: AUC_{0-12}, C_{max}, and C_{trough}

- **PK evaluations:**

A summary of steady-state PK parameter estimates for amantadine, acetylamantadine, oseltamivir and oseltamivir carboxylate will be presented. Descriptive statistics such as arithmetic mean and standard deviation will be presented for each PK parameter.

Pharmacodynamic assessments

Not applicable

Estimated total blood volume taken per subject: 231 mL.

Statistical methods

No formal sample size calculation was performed. A sample size of 18 subjects will be used based on the empirical experience for DDI studies and the exploratory nature of the study.

Data Presentation and Statistical Analysis:

For both amantadine and oseltamivir, log-transformed PK parameters AUC and C_{max} will be analyzed by a linear mixed effect model, with fixed effects from sequence, treatment, and period, and random effects from subject nested in sequence. Estimators for the treatment difference including the corresponding 90% confidence intervals will be obtained based upon the log-transformed observations. The estimators and confidence intervals will then be “back-transformed” to the original scale. The resulting 90% confidence interval of the appropriate treatment mean ratios will be used to explore the drug-drug interactions.

Assessment schedule

Study Phase	Screening	Baseline	Treatment															EOS
Visit Numbers **Treatment Period 1**	Visit 1	Visit 2 (Day -1)	Visit 3 (Day 1)				Visit 4 (Day 5)											
Visit Numbers **Treatment Period 2**		Visit 5 (Day -1)	Visit 6 (Day 1)				Visit 7 (Day 5)											
Visit Numbers **Treatment Period 3**		Visit 8 (Day -1)	Visit 9 (Day 1)				Visit 10 (Day 5)											Visit 777
Time from dose	D -21 to -2		0	2	12	14	0	0.5	1	1.5	2	3	4	6	8	10	12	12 [5]
Inclusion /Exclusion criteria	x	x [2]																
Relevant med hx/current med conditions	x	x [2]																
Demography	x																	
Physical examination	x	x [3]																x
Hepatitis and HIV screen	x																	
Alcohol test, Drug screen, Urine cotinine	x	x																
Pregnancy test	x	x																x
Meal record							x											
Drug administration record	All domiciled days																	
Study completion information																		x
Comments	As required																	
Body height	x																	
Body weight & Temperature	x	x [3]																x
Blood pressure / Pulse rate	x [4]	x [4]	x	x	x	x	x				x							x
ECG evaluation	x	x																x
Hematology, Blood chemistry, Urinalysis	x	x																x
PK blood collection			x		x		x	x	x	x	x	x	x	x	x	x	x	
Adverse Events	As required from first dose of study drug																	
Concomitant meds/Therapies	As required																	

1 Visit structure given for internal programming purpose only.
2 Review of Inclusion and exclusion criteria and current medical conditions is required only at Treatment Period 1 baseline evaluation.
3 Only at the baseline of Treatment Period 1 (Visit 2, Day -1).
4 Standing and supine blood pressure and pulse measurements required. All other readings are supine.
5 EOS assessments will be performed at 12hr of visit 10 (the last day of Treatment Period 3).

1 Background

1.1 Introduction

The possible adaptation of avian influenza into an easily transmissible virus in humans, resulting in a potential pandemic of a magnitude not seen for nearly a century, has resulted in heightened awareness of influenza. In addition, it has become clear that there are not a multitude of effective therapeutic options. Only two classes of compounds are presently licensed for treatment or prophylaxis of influenza, the ion channel inhibitors amantadine and rimantadine (active only against influenza A) and the neuraminidase inhibitors oseltamivir and zanamavir. Due to the increased resistance against amantadine of circulating seasonal influenza strains, the intrinsic resistance of most H5N1 strains, and the rapid emergence of resistance to ion channel inhibitors when they are used in vitro, amantadine is not considered to be first line agent for use in pandemic influenza resulting from human adaptation of H5N1 avian influenza. Because of its oral availability, activity against most strains of H5N1, and extensive data, oseltamivir is considered the likely drug of choice for both treatment and prophylaxis for potential pandemic influenza at this time.

There remains a concern that indiscriminate use of oseltamivir may result in the development of widespread resistance that would render it ineffective. Modeling has shown that this is more likely to occur as the result of treatment than as a result of prophylactic therapy. As some strains of H5N1 remain sensitive to amantadine, the question has arisen as to whether the use of the oseltamivir and amantadine in combination may not represent a reasonable approach to improve outcomes and to prevent the emergence of resistance. Data generated by the Webster group at St. Jude’s has now shown that there is some basis for both of these theories. First, a study of the combination in mice showed that indeed the use of this combination in H5N1 drug-sensitive viruses prevented the emergence of resistance to oseltamivir, which occurred rapidly when oseltamivir was used alone. A second study published in abstract form by this group now shows that the combination of drugs was more effective at lowering viral load and preventing clinical illness in mice infected with H5N1 than when oseltamivir was used alone. Amantadine by itself was almost completely ineffective in this model.

Other combinations of drugs used against influenza have been studied with similar results. Ribavirin and rimantadine have been shown to have a synergistic effect in vitro in MDCK cells, and ribavirin and amantadine were synergistic in vivo in mice. Rimantadine and zanamavir have been studied in vitro with promising results, and a small trial in humans revealed a potential benefit of the combination, although the trial was underpowered.

Line listings obtained from follow-up studies of amantadine (Novartis) or oseltamivir (Roche) have shown that physicians will use these drugs in combination or series in treating seriously ill influenza patients. In addition, there may actual be a theoretical reason to consider combination therapy to preserve the activity of oseltamivir if the circulating pandemic virus is fully or partially sensitive to amantadine.

For these reasons, it is sensible to produce data to evaluate the pharmacokinetics of the two drugs when used in combination, and to rule out an unexpected adverse event that may occur at a relatively high frequency. There are no theoretical considerations from knowledge of the drug metabolism pathways of either drug (noting that oseltamivir is the prodrug of oseltamivir carboxylate) that would lead one to predict any meaningful interaction. Nonetheless, the following trial is designed to provide some data in humans as to the effects of using the drugs in combination, and to assess clearance.

1.2 Summary of relevant data

A summary of relevant data is presented in Symmetrel® and Tamiflu® Package Inserts.

Pharmacokinetics of amantadine:

Amantadine is well absorbed orally. Maximum plasma concentrations are directly related to dose for doses up to 200 mg/day. It is primarily excreted unchanged in the urine by glomerular filtration and tubular secretion. Eight metabolites of amantadine have been identified in human urine. One metabolite, an N-acetylated compound, was quantified in human urine and accounted for 5-15% of the administered dose. Plasma acetylamantadine accounted for up to 80% of the concurrent amantadine plasma concentration in 5 of 12 healthy volunteers following the ingestion of a 200 mg dose of amantadine. Acetylamantadine was not detected in the plasma of the remaining seven volunteers.

After the oral administration of a single 100 mg amantadine hydrochloride capsule, several studies showed mean maximum plasma concentrations of 0.2 to 0.4 µg/mL and mean times to peak concentration of 2.5 to 4 hours. Mean half-lives ranged from 10 to 14 hours. Across other studies, amantadine plasma half-life has averaged 16 ± 6 hours (range 9 to 31 hours) in 19 healthy volunteers.

Pharmacokinetics of oseltamivir:

Oseltamivir is readily absorbed from the gastrointestinal tract after oral administration of oseltamivir phosphate and is extensively converted predominantly by hepatic esterases to oseltamivir carboxylate. At least 75% of an oral dose reaches the systemic circulation as oseltamivir carboxylate. Exposure to oseltamivir is less than 5% of the total exposure after oral dosing. Plasma concentrations of oseltamivir carboxylate are proportional to doses up to 500 mg given twice daily.

Coadministration with food has no significant effect on the peak plasma concentration (551 ng/mL under fasted conditions and 441 ng/mL under fed conditions) and the area under the plasma concentration time curve (6218 ng·h/mL under fasted conditions and 6069 ng·h/mL under fed conditions) of oseltamivir carboxylate.

Oseltamivir is extensively converted to oseltamivir carboxylate by esterases located predominantly in the liver. Neither oseltamivir nor oseltamivir carboxylate is a substrate for, or inhibitor of, cytochrome P450 isoforms. Absorbed oseltamivir is primarily (>90%) eliminated by conversion to oseltamivir carboxylate. Plasma concentrations of oseltamivir declined with a half-life of 1 to 3 hours in most subjects after oral administration. Oseltamivir carboxylate is not further metabolized and is eliminated in the urine. Plasma concentrations of

oseltamivir carboxylate declined with a half-life of 6 to 10 hours in most subjects after oral administration.

1.3 Study and dose rationale

Line listings obtained from follow-up studies of amantadine (Novartis) or oseltamivir (Roche) have shown that physicians will use these drugs in combination or series in treating seriously ill influenza patients. Therefore, it is sensible to produce data to evaluate the pharmacokinetics of the two drugs when used in combination, and to rule out an unexpected adverse event that may occur at a relatively high frequency.

Amantadine Study Dose: A 100 mg BID dose of amantadine was chosen for the study since it is the dose usually prescribed in the clinic.

Oseltamivir Study Dose: A 75 mg BID dose of oseltamivir was chosen for the study since it is the dose usually prescribed in the clinic.

The duration of the treatment period selected for this study exceeds the time to reach steady state concentration for amantadine, oseltamivir and oseltamivir carboxylate. Amantadine has the longest average plasma half-life of 16 hrs. Therefore treatment period of 5 days is adequate to achieve steady state for amantadine, oseltamivir as well as the metabolite, oseltamivir carboxylate.

2 Study purpose

In the event of a pandemic avian influenza (bird-flu), it is very likely that oseltamivir will be combined with amantadine to prevent the emergence of resistance against oseltamivir. The purpose of this study is to make limited safety and pharmacokinetic information available for the combination of amantadine and oseltamivir to health-care providers in a timely manner.

3 Objectives

3.1 Primary objectives

- To characterize the pharmacokinetics of amantadine 100 mg following twice daily administration alone or in combination with twice daily oseltamivir 75 mg in healthy volunteers.
- To characterize the pharmacokinetics of oseltamivir 75 mg following twice daily administration alone or in combination with twice daily amantadine 100 mg in healthy volunteers.

3.2 Secondary objective

- To assess the safety and tolerability of twice daily oseltamivir 75 mg and twice daily amantadine 100 mg when given alone and when given in combination.

4 Overview of study design

This is an open-label, multiple dose, randomized, three-way crossover study in healthy volunteers. A total of 18 healthy subjects will be enrolled. Dropouts may be replaced. Each subject will participate in a 21-day screening period, three baseline periods, three 5-day treatment periods, two 5 to 7-day washout periods, and a study completion evaluation.

The treatment sequence is outlined in Table 4-1.

Table 4-1 Treatment sequences

**Treatment:** Subjects will be randomized using the following scheme (with a wash-out period of 5 days between each treatment):				
**Treatment Sequence**	**Sample Size**	**Period 1**	**Period 2**	**Period 3**
A	3	Treatment 1	Treatment 2	Treatment 3
B	3	Treatment 1	Treatment 3	Treatment 2
C	3	Treatment 2	Treatment 1	Treatment 3
D	3	Treatment 2	Treatment 3	Treatment 1
E	3	Treatment 3	Treatment 1	Treatment 2
F	3	Treatment 3	Treatment 2	Treatment 1
Treatment 1 = Amantadine 100 mg BID for five days<br>Treatment 2 = Oseltamivir 75 mg BID for five days<br>Treatment 3 = [Amantadine 100 mg + Oseltamivir 75 mg] BID for five days				

Screening for this study will occur within 21 days of the first dose. Subjects will be admitted to the study site on Day -1 for baseline safety assessments and to confirm eligibility. Subjects who meet the inclusion/exclusion criteria at the first baseline will be randomized to one of the six sequences above.

During Period 1, subjects will receive the first dose of the assigned study drug(s) on Day 1 and remain domiciled for the full 5 days of each treatment period. Dosing will occur on each day between 0800 and 0830 for the morning dose(s) and between 2000 and 2030 for the evening dose(s). Pre-dose pharmacokinetic samples will be collected on the first day (Day 1) of each treatment Period. Safety assessments will be performed during the 5-day domiciled treatment period. On the evening of Day 4, subjects will fast overnight (10 to 12 hours) prior to dosing and for 4 hours after drug administration. Pharmacokinetic assessments will start pre-dose and continue up to 12 hours post dose on Day 5. Subjects will be discharged from the study center after the last pharmacokinetic and safety assessments have been performed. Subjects will return to the study site for Treatment Periods 2 and 3 after a washout period of at least 5 days but no longer than 7 days.

Serial pharmacokinetic blood sampling will occur on the last day of each Treatment Period up to 12 hours following drug administration. In addition, pre-dose samples will be collected prior to the morning and evening doses on the first day of each Treatment Period. On the serial pharmacokinetic sampling day, subjects will only receive the morning dose of study drug(s).

Subjects who wish to discontinue the study or, who are withdrawn from the study by the investigator for any reason, will be asked to complete the End of Study assessments and may be replaced.

Table 4-2 Treatment period overview

The 3 Treatment Periods will follow this schedule:

Day -1	Day 1 to 5	
Baseline	Treatment 1, 2 or 3	5-7 Days Washout Period
	Predose PK on Day 1*<br>Serial PK sampling on Day 5*	

*exact times are indicated in the assessment schedule

For a detailed outline of safety assessments, please refer to the assessment table in the synopsis section.

5 Population

The study population will comprise the following:

- Healthy males and females
- Non-smokers

All subjects who prematurely withdraw from the study must complete Study Completion evaluations.

5.1 Entry criteria

The investigator must ensure that all subjects being considered for the study meet the following inclusion and exclusion criteria. No additional exclusions should be applied by the investigator, in order that the study population will be representative of all eligible subjects.

Subjects selection is to be established by checking through all inclusion/exclusion criteria at screening and baseline of Treatment Period 1. A relevant record (e.g. checklist) must be stored with the source documentation at the study site.

The investigator or his/her deputy must promote compliance with the protocol for the duration of the study.

5.1.1 Inclusion criteria

1. Healthy male or female subjects aged 18 to 45 years, and in good health as determined by past medical history, physical examination, vital signs, electrocardiogram, and laboratory tests at screening and baseline of Treatment Period 1.
2. At Screening, and Baseline, vital signs (systolic and diastolic blood pressure and pulse rate) will be assessed after the subject has rested for at least three (3) minutes, and again when required after three (3) minutes in the standing position. Vital signs should be within the following ranges:

   oral body temperature 35.0 - 37.5°C
   systolic blood pressure 90 - 140 mm Hg
   diastolic blood pressure 50 - 90 mm Hg
   pulse rate 40 - 90 bpm

   When blood pressure and pulse are again measured after 3 minutes in a standing position, there should be no more than a 20 mm Hg drop in systolic or 10 mm Hg drop in diastolic blood pressure, nor increase in heart rate (> 20 bpm) i.e., postural hypotension.

   All subsequent blood pressure measurements should be assessed with the subject seated, unless stated otherwise in the protocol design. The cuff should be applied to the same arm for each reading.
3. Female subjects of child bearing potential must be using double-barrier local contraception, e.g., intra-uterine device plus condom, or spermicidal gel plus condom.

OR

Postmenopausal women must have no regular menstrual bleeding for at least 1 year prior to inclusion. Menopause will be confirmed by a plasma follicle-stimulating hormone (FSH) concentration of > 40 IU/L.

OR

Female subjects must have been surgically sterilized at least 6 months prior to screening. Surgical sterilization procedures must be supported with the clinical documentation made available to the sponsor, and noted in the Relevant Medical History / Current Medical Conditions section of the CRF.

4. Body mass index (BMI) must be within the range of 18 – 30 kg/m^2. For instructions and tables see **Appendix 5**. Subjects must weigh at least 50 kg to participate in this study.
5. Subjects must be able to communicate well with the investigator, to understand and comply with the requirements of the study, and understand and sign the written informed consent.

5.1.2 Exclusion criteria

Subjects meeting any of the following criteria will be excluded from entry into, or continuation in the study:

1. Smokers (use of tobacco products in the previous 3 months). Urine cotinine levels will be measured during screening for all subjects. A smoker is defined as any subject/patient who reports tobacco use or has a urine cotinine greater than 500 ng/mL

2. Female subjects who are pregnant or lactating
3. Use of any prescription drugs in the 4 week period prior to dosing, or use of over-the-counter (OTC) medication (vitamins, herbal supplements, dietary supplements) in the 2 week period prior to dosing. Paracetamol/acetaminophen and ibuprofen use is acceptable, but must be documented in the concomitant medications/significant non-drug therapies page of the CRF.
4. Participation in any clinical investigation in the 4 week period prior to dosing. A longer period should be used if required by local regulations.
5. Donation or loss of = 400 mL of blood within 8 weeks prior to first dosing, or longer if required by local regulation.
6. Significant illness within two weeks prior to dosing.
7. A past medical history of clinically significant ECG abnormalities or a family history (grandparents, parents and siblings) of a prolonged QT-interval syndrome.
8. History of autonomic dysfunction (e.g., history of fainting, orthostatic hypotension, sinus arrhythmia).
9. History of acute or chronic bronchospastic disease (including asthma and chronic obstructive pulmonary disease, treated or untreated),
10. History of clinically significant drug allergy or history of atopic allergy (asthma, urticaria, eczematous dermatitis). A known hypersensitivity to the study drug or to similar drugs.
11. Any surgical or medical condition which may alter the absorption, distribution, metabolism or excretion of drugs, or which could jeopardize a subject/patient participating in the study. The investigator should be guided by evidence of any of the following:
    - history of inflammatory bowel syndrome, gastritis, ulcers, gastrointestinal or rectal bleeding;
    - history of major gastrointestinal tract surgery such as gastrectomy, gastroenterostomy, or bowel resection;
    - history, or clinical evidence of pancreatic injury or pancreatitis;
    - clinical evidence of liver disease or liver injury as indicated by abnormal liver function tests such as ALT, AST, GGT, alkaline phosphatase, or serum bilirubin. ALT must be strictly within the reference range before inclusion, GGT and alkaline phosphatase must not exceed twice the upper limit of the reference range, serum bilirubin should not exceed 27 µmol/L (1.6 mg/dl). If the total bilirubin concentration is increased above 1.5 times the upper limit of normal, it should be differentiated into the direct and indirect reacting bilirubin;
    - history or presence of impaired renal function as indicated by clinically significantly abnormal creatinine or urea values or abnormal urinary constituents (e.g., albuminuria);
    - evidence of urinary obstruction or difficulty in urinating at screening
    - polymorphonuclears not within the range of 4000 – 11000/µL or platelets < 100000/µL at inclusion.

12. History of immunodeficiency diseases, including a positive HIV (ELISA and Western blot) test result.
13. A positive Hepatitis B surface antigen (HBsAg) or Hepatitis C test result.
14. History of drug or alcohol abuse in the 12 months prior to dosing, or evidence of such abuse as indicated by the laboratory tests conducted during the screening or baseline evaluations.

5.2 Dietary, fluid and other restrictions

During recruitment, informed consent review, and baseline period, the subjects will be informed and reminded of the following restrictions:

- No strenuous physical exercise (e.g., weight training, aerobics, football) for 7 days before dosing until after the study completion evaluation.
- No alcohol for 48 hours before dosing until after the study completion evaluation.

Intake of xanthine (e.g., caffeine) containing food or beverages must be discontinued 48 hours before dosing. Consumption of such foods and beverages (e.g., coffee, tea, soda, chocolate) is not permitted at any time while the subjects are domiciled. If a deviation occurs, it must be noted in the notes field of the CRF.

All subjects will fast for 10-12 hours prior to administration of the last dose of the study medication at each Treatment Period and will continue to fast for at least 4 hours thereafter. No fluid intake apart from the fluid given at the time of drug intake is allowed from 2 h before or after dosing. Meals should be similar in caloric content and distribution for all subjects on the days of dosing.

When mealtimes coincide with bleed times, blood will be drawn BEFORE the meal is provided.

6 Treatment

6.1 Study drugs

Study drugs must be received at the study site by a designated person, handled and stored safely and properly, and kept in a secured location to which only the investigator and designated assistants have access. Upon receipt, the study drugs should be stored according to the instructions specified on the drug labels. Clinical supplies are to be dispensed only in accordance with the protocol.

Storage conditions must be adequately monitored and appropriate temperature/humidity logs maintained as source data.

The investigator must maintain an accurate record of the shipment and dispensing of study drugs in a drug accountability ledger. Drug accountability will be noted by the field monitor during site visits and/or at the completion of the trial.

At the investigator’s site, a pharmacist will dispense and label the study medications in individual subject-specific packages according to the randomization list provided by Novartis. Appropriate documentation of the subject-specific packaging process must be maintained. Medication labels will be in the local language, will comply with the legal requirements of each country, and will include storage conditions for the drug, but no information about the subject.

All drug supplies are to be used only for this protocol and not for any other purpose. Unless specifically instructed by Novartis, the investigator must not destroy any drug labels, or any partly used or unused drug supply.

At the conclusion of the study, and, when requested during the course of the study, the investigator will provide a copy of the drug accountability ledger to the Novartis monitor.

Only after receiving written authorization by Novartis, will the investigator send all the unused and partly used drug supplies as well as the empty containers to the address provided at the time of authorization for destruction.

6.2 Treatment assignment

Randomization will be performed by Novartis Drug Supply Management using a validated system. The randomization scheme will be reviewed and approved by the Biostatistics Operation/Quality Assurance group of Novartis, and locked after approval.

For preparation of the study medication, a copy of the randomization schedule will be sent to the pharmacist at the investigator’s site or to the packaging contractor, as appropriate.

Randomization data are kept strictly confidential, and are accessible only to authorized personnel, until the time of study drug administration. Confidentiality of randomization data is required to limit the occurrence of potential bias arising from the influence that the knowledge of treatment assignment may have on the recruitment and allocation of subjects/patients.

Subjects/patients will be assigned randomization numbers, according to the randomization schedule. The randomization number becomes the definitive subject number as soon as the first dose of the respective study treatment is received.

6.3 Treatment blinding

This is an open label study.

6.4 Emergency unblinding of treatment assignment

Not applicable

6.5 Treating the subjects

6.5.1 Study drug administration

All doses prescribed and dispensed to the subject and all dose changes during the study must be recorded on the Dosage Administration Record (DAR) of the CRF.

Study medication will be administered with 240 mL of water by the study center personnel twice a day, between 0800 and 0830 in the morning and between 2000 and 2030 in the evening. Each subject's mouth must be checked to ensure that the medication was swallowed.

Subjects must be instructed not to chew the medication, but to swallow it whole. For pharmacokinetic assessment days, subjects have to rest quietly in the upright position for the 4 hours after dosing, unless performing a study assessment, e.g., ECG.

6.5.2 Permitted study drug adjustments

Changes to the doses specified in this protocol are not permitted.

6.5.3 Concomitant treatment

Administration of concomitant medication may require a subject to be discontinued.

Except for medication which may be required to treat adverse events, no medication other than study drug will be allowed from the time of the first dose of study treatment until all of the study completion evaluations have been completed.

Administration of paracetamol/acetaminophen and ibuprofen (up to 2400 mg/day) is acceptable, but must be documented. The administration of any medication (other than study drug) and significant non-drug therapies (including physical therapy and blood transfusions) administered after the subject starts study drug administration must be listed on the Concomitant medications/Significant non-drug therapies section of the CRF.

6.5.4 Recommended treatment of adverse events

Not Applicable

6.5.5 Study drug discontinuation

All subjects who discontinue study drug should be considered withdrawn from the study but should complete the study completion visit assessments. If subjects fail to return for these visits or are unable to do so, every effort should be made by the investigator to contact them or a knowledgeable informant by telephone, or by sending appropriate correspondence (e.g., certified letter) that will become part of the Investigators' file to record that efforts were made to reach the subjects.

6.5.6 Premature subject withdrawal

Subjects may voluntarily withdraw from, or be withdrawn from the study at the discretion of the investigator or the sponsor at any time. Patients must be withdrawn from the study if any of the following occur:

1. withdrawal of consent
2. adverse event(s) as judged by the investigator to be a clinically significant event. This decision shall be based upon the nature and degree of the observed abnormality

3. abnormal laboratory value(s) as judged by the investigator to be a clinically significant abnormality. This decision shall be based upon the nature and degree of the observed abnormality
4. protocol violation
5. administration of concomitant medications other than paracetamol or ibuprofen.

If such withdrawal occurs, subjects should complete the study completion visit assessments. If subjects fail to return for these visits or are unable to do so, every effort should be made by the investigator to contact them or a knowledgeable informant by telephone, or by sending appropriate correspondence (e.g., certified letter) that will become part of the Investigators' file to record that efforts were made to reach the subjects. If the subjects fail to return for visits, the investigator must determine the primary reason for a subject's premature withdrawal from the study and record this information on the Study Completion CRF page.

7 Assessments

The timing of assessments required during the study are detailed in the Study Synopsis and the Assessment Schedule. All data obtained from these assessments must be supported in the patient's source documentation. Source documentation must be available for all data entered in the CRFs. CRFs are not substitute for source documents.

Repeat evaluation or additional assessments

Should it become necessary to repeat an evaluation (e.g., ECGs, laboratory tests, vital signs, etc.), the results of the repeat evaluation should be entered on the additional assessment pages supplied with the CRFs.

When a laboratory assessment is outside the reference ranges at screening, the investigator should contact the sponsor as soon as the result of the repeat assessment becomes available. The decision on how to proceed will be made jointly by the investigator and the sponsor after review of the available safety, and will be documented in the source data/subject folder.

The additional assessment page of the CRF should be placed sequentially behind the original evaluation page. Numbering of the page should reflect the page order of the original evaluation and the number of the repeat evaluation (e.g., following an assessment on page 9, the additional assessment pages should be 9.1, 9.2, etc.), and should also reflect the date and time of additional assessment.

7.1 Background, demographic and administrative assessments

Demographics

These include date of birth, age, sex, race, height, and weight.

Relevant medical history / Current medical conditions

Relevant medical history and current medical conditions will be recorded on the respective CRF page until the start of the study drug.

AEs occurring prior to study drug administration will be reported in the Relevant Medical History section.

Physical examination

This evaluation will include an examination of general appearance, skin, neck (including thyroid), eyes, ears, nose, throat, lungs, heart, abdomen, back, lymph nodes, extremities and a basic evaluation of the nervous system.

Information about the physical examination must be included in the source documentation at the study site. Adverse events that are present prior to the start of study drug must be included in the Relevant medical history/Current medical conditions pages of the CRF. Adverse events occurring after the start of study drug administration and which meet the definition of an AE must be recorded in the Adverse Event CRF summary page.

Hepatitis screen, HIV screen

All subjects will be screened for Hepatitis B surface antigen (HBsAg). Screening for Hepatitis C will be based on the presence of HCV antibodies.

Evaluation for HIV seropositivity will be performed, and, if positive, confirmed by a second technique available at the laboratory site, e.g., Western blot or ELISA.

Appropriate subject counseling will be made available by the investigator in the event of a positive finding. Notification of state and federal authorities, as required by law, will be the responsibility of the investigator.

Alcohol test, drug screen, urine cotinine

Subjects will be tested for substances of abuse (alcohol, amphetamines, barbiturates, benzodiazepines, cannabinoids, cocaine, and opiates).

Each subject will also be tested for urine cotinine. Subjects with concentrations greater than the level indicated in the exclusion criteria will be considered smokers.

Pregnancy test

A serum test is mandatory for pregnancy tests performed at the screening or at all baseline visits. Urine pregnancy test(s) may be performed in place of serum pregnancy test(s) thereafter, including at the last visit.

Drug administration record

Date and time of dose administration will be recorded in the Dosage administration record section of the CRFs.

Meal record

For meals on days indicated in the Synopsis' Assessments and evaluations Section, the date and start time of meal consumption will be recorded in the appropriate section of the CRFs.

Study completion information

Information on the date the subject last took drug, the subject's completion or discontinuation of the study and the reason for discontinuation of the study will be recorded on the Study Completion CRF page.

Study Completion evaluations must also be performed when a subject prematurely withdraws from the study for whatever reason.

For subjects who are lost to follow-up, the investigator should show due diligence by documenting in the source documents steps taken to contact the subject, e.g., dates of telephone calls, registered letters, etc.

Comments

All comments related to study conduct will be added to the Notes field of the CRF.

7.2 Safety and tolerability assessments

Vital signs and body measurements

Body height, body weight and oral body temperature will be obtained at specified times during the study. Body weight taken at baseline of the first treatment period will be utilized for all pharmacokinetic calculations.

Systolic and diastolic blood pressure and pulse rate will be assessed after the subject has rested in the supine position for at least 3 minutes, and (if required e.g. at Screening and Baseline) again after 3 minutes in the standing position. The cuff should be applied to the same arm for each reading.

ECG evaluations

A standard 12-lead ECG will be performed. The subject's number and initials, the date and actual time of the tracing, and the study code CSYO380A2101 must appear on each page of the trace. Traces will be dated and signed by the person who performs the interpretation.

The CRFs will contain the time of ECG, the ventricular rate, PQ or PR interval, QRS duration, QT interval (uncorrected) and the overall interpretation (normal, clinically insignificant abnormality, and clinically significant abnormalities which need to be specified further).

The original ECG traces, will be archived at the study site.

Standard clinical laboratory evaluations

Screening and early morning assessment will be performed after an overnight fast.

The investigator will evaluate the clinical significance of each laboratory value which does not fall within the reference range. This decision shall be based upon the nature and degree of the observed abnormality. Values which are considered clinically significant and/or study drug related will be noted in the Comments page of the CRFs with reference to the date, study day, and hour (if applicable). The investigator may choose to repeat any abnormal result ONCE ONLY, in order to rule out laboratory error. "NCS" will be entered on the original laboratory sheet to the right of all laboratory values which are outside the reference range, but are judged "not clinically significant" and archived with the source documents. The physician making these assessments shall date and sign, or initial each laboratory report.

Clinically relevant deviations of laboratory test results occurring during, or at completion of the study must be reported and discussed with Novartis personnel. Follow-up evaluations are mandatory until their normalization or until the change is no longer clinically relevant. In case of doubt, Novartis personnel must be contacted.

A copy of each standard clinical laboratory report must accompany the corresponding CRF page. In the Comments page of the CRF a comment should be entered confirming that the value of the laboratory test/s reported outside the normal ranges was evaluated either as “NCS” or ‘Clinically Significant” by the investigator.

Hematology

The following variables will be measured:

Hemoglobin, hematocrit, WBC count (with differential as percentage or absolute value), RBC count and platelet count.

Blood chemistry

The following variables will be measured:

Albumin, alkaline phosphatase, total bilirubin, calcium, cholesterol, creatinine, CK, GGT, glucose, LDH, inorganic phosphorus, lipase, amylase, potassium, total protein, AST, ALT, sodium, triglycerides, urea and uric acid. If the total bilirubin concentration is increased above 1.5 times the upper limit of normal, direct and indirect reacting bilirubin should be differentiated.

Urinalysis

The following variables will be measured:

Specific gravity, pH, semi-quantitative "dipstick" evaluation of glucose, protein, bilirubin, ketones, leukocytes, blood. A microscopic examination including RBC, WBC, proteins, and casts will be performed **only** when dipstick evaluation is positive for WBC, or proteins, or blood. If casts are noted, the type is to be specified on the relevant CRF page. A midstream

urine sample (about 30 mL) will be obtained, in order to avoid contamination with epithelial cells and sediments, and allow proper assessments.

7.3 Efficacy assessments

Not Applicable

7.4 Pharmacokinetics

Blood collection

All blood samples will be taken by either direct venipuncture or an indwelling cannula inserted in a forearm vein. A 3 mL venous blood sample should be taken by disposable plastic syringe and dispensed into EDTA plastic collection tubes for drug assays. Vacutainers or Monovettes are acceptable alternatives. Immediately following collection, the container will be slowly inverted backwards and forwards (without shaking) several times to bring the anticoagulant into solution.

Blood Collections

- Blood collection for amantadine: At predose, 0.5, 1, 1.5, 2, 3, 4, 6, 8, 10 and 12 hr on the last day of each Treatment Period (Day 5), and pre-dose samples prior to morning and evening doses on the first day of each Treatment Period (Day 1).
- Blood collection for oseltamivir: At predose, 0.5, 1, 1.5, 2, 3, 4, 6, 8, 10 and 12 hr on the last day of each Treatment Period (Day 5), and pre-dose samples prior to morning and evening doses on the first day of each Treatment Period (Day 1).

Plasma (~1.5 mL) should be separated by centrifugation (~1000 g and 4°C for 10 minutes) within 60 minutes of blood collection, and the supernatant plasma carefully transferred into two separate 2-mL screw-cap, polypropylene vials. The plasma should be stored in the tubes labeled as specified in Appendix 3. If centrifugation is not performed immediately after blood collection, the blood must be stored in a water-ice bath or in a refrigerator at 4°C until centrifugation.

Plasma samples should be immediately stored, in an upright position at or below -15°C for amantadine or -70°C for oseltamivir. The temperature of the freezers must be maintained and monitored.

The set of plasma samples designated for amantadine will be shipped frozen in dry ice to CEDRA Corporation in Austin, TX. The second set of plasma samples designated for oseltamivir will shipped frozen in dry ice to Bioanalytical Systems Ltd. in the UK. See below for instructions for shipment.

Analytes

Amantadine media and methods: amantadine and acetylamantadine plasma concentrations will be determined using a validated liquid chromatography-tandem mass spectrometry (LC-MS/MS) method with an LLOQ of approximately 5.00 ng/mL for each analyte.

Oseltamivir media and methods: oseltamivir and oseltamivir carboxylate plasma concentrations will be determined using a validated high–performance liquid chromatography/mass spectrometry method with LLOQs of approximately 1 ng/mL and 10 ng/mL respectively.

The exact clock time of dosing, as well as actual sample collection date and time will be entered on the PK blood collection summary page of the CRFs. Sampling problems will be noted in the Comments section of the CRFs.

7.5 PK sample handling, labeling and shipment instructions

7.5.1 Sample labeling

Plasma tubes

The plasma samples taken for the analysis of the various analytes will be labeled according to the following example:

Study Code: CSYO380A2101
Subject Number: 5101 (or 6101 if replaced)
Sample Number: Sample 101 (see Appendix 3 for numbering system)
Required Time: 0 h

The investigator will provide printed labels containing all of the above information.

The exact clock time of dosing, as well as actual sample collection date and time will be entered on the PK blood collection summary page of the CRF. Any sampling problems will be noted in the relevant field of the CRF.

7.5.2 Shipment of pharmacokinetic samples

For each shipment, all pharmacokinetic samples are to be entered in the shipping log reported in the ‘PK Samples Shipping Log’ page shown in Appendix 4. The original document will be retained at the site in the Investigator’s file, and a copy of the PK samples shipping log page must be included with the package of PK-samples shipped.

All pharmacokinetic specimens will be kept at the temperature specified above until shipment. Unless instructed otherwise, the samples will be packed carefully with suitable packing material and dry ice to keep them frozen. Laboratory analyses of specimens collected in this study for determination of amantadine and metabolite concentrations will be assigned by the Novartis Bioanalytics Section as noted below.

Sample shipment instructions

Instructions for shipment of Amantadine and acetylamantadine samples:

Plasma samples for amantadine and acetylamantadine analysis should be shipped on dry ice (use about 10 kg of dry ice per box) to:

Sample Control – Stephanie Olofson

CEDRA Corporation

8609 Cross Park Drive

Austin, TX 78754

Phone: 512 834 7766

Fax: 512 834 1165

All shipments should be sent (Monday through Wednesday **only**) by a carrier guaranteeing overnight delivery. The following items should be considered:

- Advise the carrier of the type of service desired, need for personalized door-to-door pickup, and delivery guaranteed within 24 hours.
- Advise the carrier of the nature of the shipment's contents (human biological specimens) and label the package accordingly.
- Indicate Novartis drug code "SYO380" and Study No. "CSYO380A2101"on the face of the parcel to be shipped. The parcel also must carry a "dangerous goods" label because of the dry ice (labels supplied by the courier).
- The carrier must be asked to store the parcel(s) in a freezer if shipment is delayed and to replace exhausted dry ice before transportation continues.
- Shipping reservations should be made to allow delivery to CEDRA Corporation before 16:00 (4 pm) local time Monday to Thursday and before 11:00 (11 am) local time on Friday. Shipments should not be sent between Thursday and Sunday, to prevent arrival over the weekend.

Insert the inventory reported in Appendix 4, report all specimens enclosed, including the date, Specimen # and time point associated with each specimen.

- Clearly indicate any missing specimens.
- Copies of the completed PK blood collection CRF forms if using paper CRFs, and copy of the shipping log must be included.

Notify the Bioanalyst that shipment is scheduled. Call 512-834-7766 or E-mail **solofson@cedracorp.com** with the following details:

- To whom the shipment is addressed, the study number, carrier and the shipping form number (or equivalent airbill number)
- The sender's name, telephone number and alternative contact personnel
- The time and date of shipping and approximate time of arrival
- The total number of cartons and unit weight of each carton

Also notify the Clinical Trial Leader at Novartis when a shipment has been scheduled.

Instructions for shipment of oseltamivir and metabolites samples:

All pharmacokinetic specimens will be kept at the temperature specified above until shipment.

Samples have to be packed according to the ICAO/IATA-Packing-Instructions in an insulated box. To guarantee that the samples remain deep frozen during transport, use about **10 kg of dry ice** per box which will keep the samples frozen during the whole duration of the transport (air freight). Send the parcel to the following.

A copy of the shipping log located in the appendix of this protocol must be included with the shipment.

Ship to:

Attn: Neil Doleman
Bioanalytical Systems Ltd.
Buildings 26-28
Stoneleigh Deer Park
Stareton, Kenilworth
Warwickshire CV8 2LQ
UK

Phone: +44 24 76 639574
Fax: +44 24 76 639568
Email: ndoleman@bioanalytical.com

A copy of the shipping log provided in the appendix of this protocol must be included with the shipment.

Please notify the addressee in advance of the shipment and indicate:

- Number of the airbill
- The time and date of shipping and approximate time of arrival
- Flight Number
- To whom the shipment is addressed, the study number, carrier and the shipping form number (or equivalent airbill number)
- The sender’s name, telephone number and alternative contact personnel
- The total number of cartons and unit weight of each carton

Also notify the study leader at Novartis when a shipment has been scheduled.

The samples should be sent at the beginning of a week in order to arrive no later than Thursday.

7.5.3 Analytical method(s)

Amantadine media and methods: amantadine and acetylamantadine plasma concentrations will be determined using a validated liquid chromatography-tandem mass spectrometry (LC-MS/MS) method with an LLOQ of approximately 5.00 ng/mL for each analyte.

Oseltamivir media and methods: oseltamivir and oseltamivir carboxylate plasma concentrations will be determined using a validated high–performance liquid

chromatography/mass spectrometry method with LLOQs of approximately 1 ng/mL and 10 ng/mL respectively.

7.6 Pharmacodynamic assessments

Not Applicable

7.7 Biomarkers

Not Applicable

7.8 Tolerability

Not Applicable

8 Safety monitoring

8.1 Reporting of adverse events

8.1.1 Definitions

Information about all adverse events, whether volunteered by the subject, discovered by investigator questioning, or detected through physical examination, laboratory test or other means, will be collected and recorded on the Adverse Events summary page of the CRFs and followed as appropriate.

Adverse events

An adverse event is the appearance or worsening of any undesirable sign, symptom, or medical condition occurring after starting the study drug even if the event is not considered to be related to study drug. Study drug includes the investigational drug under evaluation and the comparator drug or placebo that is given during any phase of the trial. Medical conditions/diseases present before starting study drug are only considered adverse events if they worsen after starting study drug. **Abnormal laboratory values or test results constitute adverse events only if they induce clinical signs or symptoms, are considered clinically significant, or require intervention.**

AEs are recorded on the Adverse Events summary page of the CRF under the signs, symptoms, or diagnosis associated with them. The sponsor should always be notified of the abnormality of test results in a timely fashion.

The occurrence of adverse events should be sought by non-directive questioning of the patient at each visit during the study. Adverse events also may be detected when they are volunteered by the subject during or between visits or through physical examination, laboratory test, or other assessments. As far as possible, each adverse event should be evaluated to determine:

- the severity grade (mild, moderate, severe) or (grade 1-4) (delete 1 of the 2 options)
- its relationship to the study drug(s) (suspected/not suspected)

- its duration (start and end dates or if continuing at final exam)
- action taken (no action taken; study drug dosage adjusted/temporarily interrupted; study drug permanently discontinued due to this adverse event; concomitant medication taken; non-drug therapy given; hospitalization/prolonged hospitalization)
- whether it constitutes a serious adverse event (SAE)

An SAE is defined as an event which:

- is fatal or life-threatening
- results in persistent or significant disability/incapacity
- constitutes a congenital anomaly/birth defect
- requires inpatient hospitalization or prolongation of existing hospitalization, unless hospitalization is for:
    - routine treatment or monitoring of the studied indication, not associated with any deterioration in condition (specify what this includes)
    - elective or pre-planned treatment for a pre-existing condition that is unrelated to the indication under study and has not worsened since the start of study drug
    - treatment on an emergency outpatient basis for an event not fulfilling any of the definitions of a SAE given above and not resulting in hospital admission
    - social reasons and respite care in the absence of any deterioration in the subject's general condition
- is medically significant, i.e., defined as an event that jeopardizes the subject or may require medical or surgical intervention to prevent one of the outcomes listed above

Unlike routine safety assessments, SAEs are monitored continuously and have special reporting requirements; see Section 9.

All adverse events should be treated appropriately. Such treatment may include changes in study drug treatment including possible interruption or discontinuation, starting or stopping concomitant treatments, changes in the frequency or nature of assessments, hospitalization, or any other medically required intervention. Once an adverse event is detected, **it should be followed until its resolution**, and assessment should be made at each visit (or more frequently, if necessary) of any changes in severity, the suspected relationship to the study drug, the interventions required to treat it, and the outcome.

Information about common side effects already known about the investigational drug can be found in the Investigator Brochure or will be communicated between IB updates in the form of Investigator Notifications. This information will be included in the subject informed consent and should be discussed with the subject during the study as needed.

8.1.2 Instructions for completing adverse event case report forms

Examples of the severity grade, relationship to study drug and actions taken, as presented in the case report form, are provided below.

The severity grade of an adverse event provides a qualitative assessment of the extent or intensity of an adverse event, as determined by the investigator or as reported by the subject. The severity grade does not reflect the clinical seriousness of the event, only the degree or extent of the affliction or occurrence (e.g. severe nausea, mild seizure), and does not reflect the relationship to study drug.

Severity grade for an adverse event

1 = Mild	Symptom barely noticeable to subject; does not influence performance or functioning. Prescription drug not ordinarily needed for relief of symptom, but may be given because of personality of subject.
2 = Moderate	Symptom of a sufficient severity to make subject uncomfortable; performance of daily activities influenced; subject is able to continue study; treatment for symptom may be needed.
3 = Severe	Symptom caused severe discomfort; may be of such severity that subject cannot continue participation in study; severity may cause cessation of treatment with study drug; treatment for symptom may be given and/or subject hospitalized.

The relationship between the administration of study drug and the occurrence of the adverse event is described as belonging to one of only two categories, either suspected by the investigator or not suspected by the investigator.

Relationship of adverse events to study drug

0 = Not suspected	The temporal relationship of the clinical event to study drug administration makes **a causal relationship unlikely**, or other drugs, therapeutic interventions or underlying conditions provide a sufficient explanation for the observed event.
1 = Suspected	The temporal relationship of the clinical event to study drug administration makes a **causal relationship possible**, and other drugs, therapeutic interventions or underlying conditions do not provide a sufficient explanation for the observed event.

The actions taken in response to an adverse event are described on a numerical scale, from 0 to 5 that cover the various possibilities. One or more of these can be selected:

Action taken in response to an adverse event

0 = No action taken

1 = Study drug dosage adjusted / temporarily interrupted

2 = Study drug permanently discontinued

3 = Concomitant medication taken

4 = Non-drug therapy given

5 = Hospitalization / prolonged hospitalization

8.2 Recommended treatment for known AEs

Not applicable

8.3 Data Monitoring Board

Not applicable

9 Serious adverse events reporting

To ensure subject safety, every SAE, regardless of suspected causality, occurring **after the subject begins taking study drug and until 4 weeks after the patient has stopped study participation** must be reported to Novartis within 24 hours of learning of its occurrence.

Any SAEs experienced after this 4-week period should only be reported to Novartis if the investigator suspects a causal relationship to the study drug.

Recurrent episodes, complications, or progression of the initial SAE must be reported as follow-up to the original episode within 24 hours of the investigator receiving the follow-up information. An SAE occurring at a different time interval or otherwise considered completely unrelated to a previously reported one should be reported separately as a new event.

Information about all SAEs is collected and recorded on the Serious Adverse Event Report Form. The investigator must assess the relationship to study drug, complete the SAE Report Form in English, and send the completed, signed form by fax within 24 hours to the local Novartis Clinical Safety & Epidemiology Department. The telephone and fax number of the contact persons in the local department of Clinical Safety and Epidemiology, specific to the site, are listed on page 2 of this protocol. The original copy of the SAE Report Form and the fax confirmation sheet must be kept with the case report form documentation at the study site.

Follow-up information is sent to the same person to whom the original SAE Report Form was sent, using a new SAE Report Form stating that this is a follow-up to the previously reported SAE and giving the date of the original report. Each re-occurrence, complication, or progression of the original event should be reported as a follow-up to that event even if it occurs at a different time interval. The follow-up information should describe whether the event has resolved or continues, if and how it was treated, whether the blind was broken or not, and whether the subject continued or withdrew from study participation.

If the SAE is not previously documented in the Investigator's Brochure or Package Insert (new occurrence) and is thought by the sponsor to be related to the Novartis study drug, a Clinical Safety & Epidemiology Department associate may urgently require further information from the investigator for Health Authority reporting. Novartis may need to issue an Investigator Notification (IN), to inform all investigators involved in any study with the same drug that this SAE has been reported.

9.1 Reporting procedures for SAEs

A SAE must be **reported to Novartis Safety and Epidemiology (CS&E) within 24 hours** of the occurrence of the event by Fax. The local CS&E fax numbers are listed on **page 2 of this protocol**.

The investigator must also **inform one of the responsible persons** of Novartis Exploratory Clinical Development **as soon as possible by telephone** (as **listed on page 2 of the Study Protocol**) of the occurrence of an SAE.

Questions pertaining to a specific serious adverse event occurring in a study subject should be directed to the Exploratory Clinical Development contact persons. Questions pertaining to the fax transmission of a SAE Report Form should be directed to the local Clinical Safety and Epidemiology (CS&E) Department to whom the original form was sent.

In close collaboration with the investigator, Novartis Exploratory Clinical Development will ensure that the SAE reporting form is completed and faxed by the investigator to the local Novartis Clinical Safety and Epidemiology (CS&E) Department. The SAE Report Form must be completed in English and assess the relationship to study drug.

9.2 Pregnancies

To ensure subject safety, each pregnancy in a subject (or patient) on study drug must be reported to Novartis within 24 hours of learning of its occurrence. The pregnancy should be followed to determine outcome, including spontaneous or voluntary termination, details of the birth, and the presence or absence of any birth defects, congenital abnormalities, or maternal and/or newborn complications.

Pregnancy should be recorded on a Clinical Trial Pregnancy Form and reported by the investigator to the local Novartis Clinical Safety & Epidemiology Department. Pregnancy follow-up should be recorded on the same form and should include an assessment of the possible relationship to the Novartis study drug of any pregnancy outcome. Any SAE experienced during pregnancy must be reported on the SAE Report Form.

10 Data review and database management

10.1 Site monitoring

Before a study is initiated (e.g., at a study site initiation visit or at an investigator's meeting) a Novartis representative will review the protocol and CRFs with the investigators and study staff.

Case report forms do not constitute source documentation, and all data entered in the CRFs must be traceable to an original source record (electronic or paper) either as part of the electronic database or in the subject's file.

During the study the study monitor may visit the site to review the implementation of the center's processes associated with completion, consistency, and accuracy of entries on the CRFs, source data verification, QC of entries into the CRFs, progress of enrollment,

adherence to the protocol and to Good Clinical Practices, and to ensure that study medication is being properly stored, dispensed, and accounted for according to protocol specifications. The investigator and key study personnel must be available to assist the study monitor during these visits.

The investigator must maintain source documents for each subject in the study, consisting of case and visit notes containing demographic and medical information, laboratory data, electrocardiograms, and the results of any other tests or assessments. **All information on the CRFs must be traceable to a source record (e.g., the subject's/patient's electronic case record and/or conventional study file)**. The investigator must also keep a copy of the signed informed consent form.

No information in source documents about the identity of the subjects will leave the study center.

The investigator (and/or his or her staff) is responsible for completing the CRFs and the study monitor is responsible for reviewing them and for clarifying and assisting in resolving any data queries. For paper CRFs, the completed and corrected CRFs will be sent for data processing, as arranged by the study monitor. A copy of the CRF is retained by the investigator, who must ensure that they are stored with other study documents, such as the protocol, the Investigators Brochure, protocol amendments, and any other critical regulatory documents, in a secure place.

10.2 Data collection and retention of documents

Data on subjects collected on CRFs during the study will be documented in an anonymous fashion and the subject will only be identified by the subject number, and by his/her initials if also required. If, as an exception, it is necessary for safety or regulatory reasons to identify the subject, both Novartis and the investigator are bound to keep this information confidential.

Designated investigator staff must enter the information required by the protocol onto the Novartis CRFs that are printed on 3-part, non-carbon-required paper. Study monitors will review the CRFs for completeness and accuracy and instruct site personnel to make any required corrections or additions. The CRFs are forwarded to Novartis by the investigational site, one copy being retained at the investigational site. Once the CRFs are received by Novartis, their receipt is recorded, the original copy is placed in Central Files, and the non-carbon-required copy is forwarded to the responsible medical data management staff for processing.

Essential documents, as listed below, must be retained by the investigator for as long as needed to comply with national and international regulations (generally 2 years after discontinuing clinical development or after the **latest** marketing approval). Novartis will notify the investigator(s)/institution(s) when the study-related records are no longer required. The investigator agrees to adhere to the document retention procedures by signing the protocol. Essential documents include:

1. IRB/IEC approvals for the study protocol and all amendments
2. All source documents and laboratory records

3. CRF basis
4. Subjects' informed consent forms
5. CVs of the investigator and other key study personnel
6. Laboratory certification and reference ranges
7. Investigator's and co-investigators' Financial Disclosure
8. FDA form 1572 (required for trials conducted in the US)
9. Any other pertinent study document, including pharmacy documents

10.3 Auditing procedures

In addition to the routine monitoring procedures, a Clinical Quality Assurance Unit exists within Novartis. This unit conducts audits of Exploratory Clinical Development activities in accordance with internal SOPs to evaluate compliance with the principles of Good Clinical Practice. A regulatory authority may also wish to conduct an inspection (during the study or after its completion). If an inspection is requested by a regulatory authority, the investigator must inform Novartis immediately that this request has been made.

10.4 Database management and quality control

Data from CRFs are entered centrally into the study database by Novartis Data Management staff using single data entry with electronic verification.

Subsequently, the entered data are systemically checked by Data Management staff , using error messages printed from validation programs and database listings. Obvious errors are corrected by Data-Management personnel. Other errors or omissions are entered on Data Query Forms are kept with the CRFs at the investigator site, and a copy is sent to Novartis so the resolutions can be entered centrally into the database. Quality control audits of all key safety and efficacy data in the database are made prior ro locking the database.

Concomitant medications entered into the database will be coded using the WHO Drug Reference List, which employs the Anatomical Therapeutic Chemical classification system. Medical history/current medical conditions and adverse events will be coded using the Medical dictionary for regulatory activities (MedDRA) terminology.

11 Data analysis

Analysis of the data will be under the direction of Novartis personnel.

11.1 Populations for analysis

All subjects with evaluable/complete PK data will be included in the PK data analysis.

The Safety Population will consist of all subjects that received at least one dose of study drug with at least one post-baseline safety assessment. Subjects will be analyzed according to treatment received.

11.2 Sample size determination

No formal sample size calculation will be performed. A sample size of 18 subjects will be used based on the empirical experience for DDI studies and the exploratory nature of the study. Since the variability of the pk parameters of both amantadine and oseltamivir is unknown, power was calculated at various levels of CV (intra-subject variability). The table below gives the power that the 90% CI of the treatment mean ratio falls between 80% and 125% when there is no actual difference between treatments.

CV	POWER
0.1	99%
0.2	88%
0.3	38%
0.4	7%

The above calculations were performed using the nQuery Advisor 5.0.

On the other hand, for a potential adverse event with the incidence rate 10%, we are 85% sure that at lease one such adverse event will appear among 18 subjects.

11.3 Power for analysis of critical secondary variables

Not Applicable

11.4 Subjects demographics/other baseline characteristics

All data for background and demographic variables such as age, weight, height, gender and race will be listed by treatment sequence and subject. Summary statistics will be provided by treatment sequence.

Relevant medical history, current medical conditions, results of laboratory screens, drug tests and any other relevant information will be listed by treatment sequence and subject

11.5 Safety and tolerability data analysis

All subjects who received at least one treatment will be included in the safety and tolerability evaluation.

11.5.1 Safety and tolerability variables

Vital signs

All vital signs will be listed by treatment group and subject and if ranges are available abnormalities (and relevant orthostatic changes) will be flagged. Summary statistics will be provided by treatment group.

ECG evaluations

All ECG data will be listed by period, group and subject and if ranges are available abnormalities will be flagged. Summary statistics will be provided by treatment group.

Standard clinical laboratory evaluations

All laboratory data will be listed by treatment group and subject and if ranges are available abnormalities will be flagged. Summary statistics will be provided by treatment group.

Adverse events

All information obtained on adverse events will be displayed by treatment group and subject.

Concomitant medications / Significant non-drug therapies

All concomitant therapies will be listed by treatment sequence and subject.

11.6 Pharmacokinetic data analysis

All completed subjects with quantifiable pharmacokinetic (PK) measurements will be included in the pharmacokinetic data analysis.

Pharmacokinetic variables

For standard pharmacokinetic abbreviations and definitions see the list provided at the beginning of this protocol.

The following pharmacokinetic parameters will be determined using non-compartmental method(s):

Primary PK Parameters: Steady state PK parameters: AUC_{0-12}, C_{max}, and C_{trough}

Biofluid concentrations will be expressed in mass per volume units. All concentrations below the limit of quantification or missing data will be labeled as such in the concentration data listings. Concentrations below the limit of quantification will be treated as zero in summary statistics and for the calculation of pharmacokinetic parameters.

Descriptive statistics of pharmacokinetic parameters will include mean, SD, and CV, min and max. Pharmacokinetic parameters will be determined using non-compartmental method(s) using WinNonlin Version 5.0.1.

Statistical methods for pharmacokinetic analyses

For both amantadine and oseltamivir, log-transformed PK parameters AUC_{0-12}, C_{max} and C_{trough} will be analyzed by a linear mixed effect model, with treatment as a fixed factor and subject as a random factor. The resulting 90% confidence interval of the appropriate treatment mean ratios will be used to examine the drug-drug interactions.

11.7 Pharmacodynamic data analysis

Not applicable.

11.8 Pharmacokinetic-pharmacodynamic data analysis

Not applicable.

11.9 Biomarkers analysis

Not Applicable

11.10 Health-related Quality of Life

Not Applicable

11.11 Interim analysis

No interim analysis has been planned.

12 References (Available upon request)

13 Responsible study personnel

Novartis study personnel

**Study Leader**<br>**Exploratory Clinical Development**	**Ahmed Amer**<br>**Novartis Pharmaceuticals Corporation**<br>**One Health Plaza 435/1172C**<br>**East Hanover, NJ 07936-1080**<br>**USA**<br>**Tel. Business +1 862 778 1333**<br>**Fax. Business +1 973 781 2077**
**Pharmacology Expert**<br>**Exploratory Clinical Development**	**Thomas Evans**<br>**Novartis Institutes for BioMedical Research, Inc.**<br>**400 Technology Square**<br>**Cambridge, MA 02139**<br>**Tel. Business +1 617 871 7698**<br>**Fax. Business +1 617 871 7629**<br>**Tel. Mobile phone +1 617 230 6589**
**Lead Pharmacokineticist**<br>**Exploratory Development**	**Sandip Roy**<br>**Novartis Pharmaceuticals Corporation**<br>**One Health Plaza**<br>**East Hanover, NJ 07936**<br>**Tel. Business +1 862 778 0015**
**Study Biostatistician**<br>**Biostatistics and Statistical Reporting**	**Marcia Wang**<br>**Novartis Pharmaceuticals Corporation**<br>**One Health Plaza**<br>**East Hanover, NJ 07936**<br>**Tel. Business +1 862 778 1029**
**Data Manager**<br>**Electronic Data Management**	**Anne Bone**<br>**Novartis Horsham Research Centre**<br>**Wimblehurst Road**<br>**GB- Horsham, West Sussex RH12 5AB**<br>**UNITED KINGDOM**<br>**Tel. Business +44 1403 323033**<br>**Fax. Business +44 1403 323054**

Addresses for sample shipment

**Bioanalyst: Amantadine / acetylamantadine**	**Sample Control – Stephanie Olofson**<br>**CEDRA Corporation**<br>**8609 Cross Park Drive**<br>**Austin, TX, 78754**<br>**Tel. Business 512 834 7766**<br>**Fax. Business 512 834 1165**
**Bioanalyst: Oseltamivir/oseltamivir carboxylate:**	**Bioanalytical Systems Ltd.- Neil Doleman**<br>**Warwickshire CV8 2LQ Buildings 26-28**<br>**Stoneleigh Deer Park, Stareton**<br>**Kenilworth, UK**<br>**Tel. Business +44 24 76 516848**<br>**Fax. Business +44 24 76 639568**

External investigators

**Principal Investigator**	**Name: Dennis Morrison**<br>**Center's Name: Bio-Kinetic Clinical Applications, Inc.**<br>**Address: 1816 W. Mt. Vernon**<br>**Springfield, MO 65802, USA**<br>**Tel 417 831 0456**<br>**Fax 417 831 0778**
**Clinical Safety Laboratory**	**Name: David Smid**<br>**Laboratory's Name: Cox Walnut Lawn**<br>**Address: 1000 E Walnut Lawn**<br>**Springfield, MO 65807, USA**<br>**Tel**<br>**Fax**
**Clinical Safety Laboratory**	**Name: David Smid**<br>**Laboratory's Name: Cox Medical Center South Lab**<br>**Address: 3801 S National**<br>**Springfield, MO 65802, USA**<br>**Tel**<br>**Fax**

14 Appendix 1: Administrative procedures

14.1 Regulatory and ethical compliance

This clinical study was designed and shall be implemented and reported in accordance with the ICH Harmonized Tripartite Guidelines for Good Clinical Practice, with applicable local regulations (including European Directive 2001/83/EC and US Code of Federal Regulations Title 21), and with the ethical principles laid down in the Declaration of Helsinki.

14.2 Responsibilities of the investigator and IRB/IEC/REB

The protocol and the proposed informed consent form must be reviewed and approved by a properly constituted Institutional Review Board/Independent Ethics Committee/Research Ethics Board (IRB/IEC/REB) before study start. A signed and dated statement that the protocol and informed consent have been approved by the IRB/IEC/REB must be given to Novartis before study start. Prior to study start, the investigator is required to sign a protocol signature page confirming his/her agreement to conduct the study in accordance with these documents and all of the instructions and procedures found in this protocol and to give access to all relevant data and records to Novartis monitors, Novartis Clinical Quality Assurance representatives, and regulatory authorities as required.

14.3 Informed consent

Eligible subjects may only be included in the study after providing written (witnessed, where required by law or regulation), IRB/IEC/REB-approved informed consent, or, if incapable of doing so, after such consent has been provided by a legally acceptable representative of the subject. Informed consent must be obtained before conducting any study-specific procedures (i.e., all of the procedures described in the protocol). The process of obtaining informed consent should be documented in the subject source documents.

Subject information and consent forms if generated by the investigator must be approved by the sponsor prior to submission to the Ethics Committee (IEC)/Institutional Review Board (IRB). A copy of the subject information and consent forms approved by the IEC/IRB must be forwarded to Novartis after IRB/IEC approval.

The informed consent form must be submitted by the investigator with the protocol for IRB/IEC approval. Any changes to the agreed upon consent form must be approved by Novartis before submission to the IRB/IEC, and a copy of the approved version must be provided to the Novartis monitor after IRB/IEC approval.

Eligible subjects may only be included in the study after providing written (witnessed, where required by law or regulation), IRB/IEC-approved informed consent, or, if incapable of doing so, after such consent has been provided by a legally authorized representative of the subject. The process of obtaining informed consent should be documented in the subject source documents.

14.4 Informed consent procedures

The informed consent should be given by means of a standard written statement, presented in non-technical language, and in a language that the subject reads and understands. The subject should read and consider the statement before signing and dating it, and should be given a copy of the signed document. No subject can enter the study before his/her informed consent has been obtained.

The investigator must explain to each subject (or legally authorized representative) the nature of the study, its purpose, the procedures involved, the expected duration, the potential risks and benefits involved and any discomfort it may entail. Each subject must be informed that participation in the study is voluntary and that he/she may withdraw from the study at any time and that withdrawal of consent will not affect his/her subsequent medical treatment or relationship with the treating physician.

Information about common side effects already known about the investigational drug can be found in the Investigator Brochure (IB) or will be communicated between IB updates in the form of Investigator Notifications. This information will be included in the subject informed consent and should be discussed with the subject during the study as needed.

14.5 Amendments to the protocol

Any change or addition to the protocol, other than administrative ones, can only be made in a written protocol amendment that must be approved by Novartis, Health Authorities where required, and the IRB/IEC/REB. Only amendments that are required for subject safety may be implemented prior to IRB/IEC/REB approval.

Notwithstanding the need for approval of formal protocol amendments, the investigator is expected to take any immediate action required for the safety of any subject included in this study, even if this action represents a violation of the protocol. In such cases, Novartis should be notified of this action and the IRB/IEC/REB at the study site should be informed within 10 working days.

14.6 Administrative Amendment

Changes to the protocol affecting only administrative aspects of the study do not require formal protocol amendments or IRB/IEC approval but the IRB/IEC must be kept informed of such administrative changes. Examples of administrative changes not requiring formal protocol amendments and IRB/IEC approval that can be treated as administrative amendments include:

1. changes in the staff used to monitor trials (e.g. Novartis staff versus a CRO)
2. minor changes in the packaging or labeling of study drug.
3. changes in shipping address for CRFs

14.7 Discontinuation of the study

Novartis reserves the right to discontinue this study under the conditions specified in the clinical trial agreement.

14.8 Publication of results

Any formal presentation or publication of data from this trial will be considered jointly between the investigator and Novartis, as indicated in the contract agreement undersigned by both parties.

15 Appendix 2: Treatment assignment

Randomization numbers	Replacement numbers
5101 – 5118	6101 – 6118

16 Appendix 3: Blood log

Blood Log: Time schedule for blood sampling for safety and pharmacokinetic (PK), assessments

Study phase	Time		Safety (mL)	Amantadine *Number*	Amantadine (mL)	Oseltamivir *Number*	Oseltamivir (mL)
**Screening**	**- 21 to -2**	**d**	**15**				
**Treatment Periods 1 - 3**							
Baseline	-1	d	15				
Predose: Day 1	0	h		101, 201	3	301, 401	3
	12	h		102, 202	3	302, 402	3
Predose: Day 5	0	h		103, 203	3	303, 403	3
Postdose: Day 5	0.5	h		104, 204	3	304, 404	3
	1	h		105, 205	3	305, 405	3
	1.5	h		106, 206	3	306, 406	3
	2	h		107, 207	3	307, 407	3
	3	h		108, 208	3	308, 408	3
	4	h		109, 209	3	309, 409	3
	6	h		110, 210	3	310, 410	3
	8	h		111, 211	3	311, 411	3
	10	h		112, 212	3	312, 412	3
	12	h		113, 213	3	313, 413	3
**End of Study (EOS)**			**15**				
Total					39		39
	Treatment 1 total:		54				
	Treatment 2 total:		54				
	Treatment 3 total:		93				
Total all periods + Screening + EOS			231				

Key to numbering system of blood collection samples:
100 Series: Treatment 1: Amantadine samples
200 Series: Treatment 3: Amantadine samples
300 Series: Treatment 2: Oseltamivir samples
400 Series: Treatment 3: Oseltamivir samples

Treatment 1 = Amantadine 100 mg BID for five days
Treatment 2 = Oseltamivir 75 mg BID for five days
Treatment 3 = [Amantadine 100 mg + Oseltamivir 75 mg] BID for five day

17 Appendix 4: PK samples shipping log

Make copies as needed

Compound: **SYO380_** Trial Study Number: **CSYO380A2101**

Shipment No. _______ Page ___of ___

Sender: ________________
Street address ________________
City, Country ________________
Tel. No. ________________
Fax No. ________________

Ship to :
CEDRA Corporation, Attn: Stephanie Olofson
8609 Cross Park Dr
Austin, TX, 78754
Tel. (001) 512 834 7766
Fax (001) 512 834 1165

Date of Shipment: ________________

**Samples' Matrix:**	**Subject ID No.**	**Treatment Period No.**	**Number of samples**	**Remarks** (Indicate if any sample is missing or the volume is less then what scheduled)
Plasma	*5101*	*1*	*13*	

Total number of samples shipped: ___________

Signature: ______________________________ Date ________

Make copies as needed

Compound: **SYO380** Trial Study Number: **CSYO380A2101**

Shipment No. _______ Page ___of ___

Sender: ____________________

Street address ____________________

City, Country ____________________

Tel. No. ____________________

Fax No. ____________________

Ship to :

Bioanalytical Systems Ltd., Attn: Neil Doleman
Warwickshire CV8 2LQ Buildings 26-28
Stoneleigh Deer Park, Stareton
Kenilworth, UK
Tel. +44 24 76 516848
Fax +44 24 76 639568

Date of Shipment: ____________________

**Samples' Matrix:**	**Subject ID No.**	**Treatment Period No.**	**Number of samples**	**Remarks**<br>(Indicate if any sample is missing or the volume is less then what scheduled)
Plasma	*5101*	*1*	*13*	

Total number of samples shipped: ____________

Signature: ______________________________ Date ________

18 Appendix 5 Determination of body weight by height criteria

18.1 Determination of Body Mass Index (weight[kg] / height[m]^2)

height (m)	46	48	50	52	54	56	58	60	62	64	66	68	70	72	74	76	78	80	82	84	86	88	90	92	94
1.44	22.2	23.1	24.1	25.1	26.0	27.0	28.0	28.9	29.9	30.9	31.8	32.8	33.8	34.7	35.7	36.7	37.6	38.6	39.5						
1.46	21.6	22.5	23.5	24.4	25.3	26.3	27.2	28.1	29.1	30.0	31.0	31.9	32.8	33.8	34.7	35.7	36.6	37.5	38.5	39.4					
1.48	21.0	21.9	22.8	23.7	24.7	25.6	26.5	27.4	28.3	29.2	30.1	31.0	32.0	32.9	33.8	34.7	35.6	36.5	37.4	38.3	39.3				
1.50	20.4	21.3	22.2	23.1	24.0	24.9	25.8	26.7	27.6	28.4	29.3	30.2	31.1	32.0	32.9	33.8	34.7	35.6	36.4	37.3	38.2	39.1			
1.52	19.9	20.8	21.6	22.5	23.4	24.2	25.1	26.0	26.8	27.7	28.6	29.4	30.3	31.2	32.0	32.9	33.8	34.6	35.5	36.4	37.2	38.1	39.0		
1.54	19.4	20.2	21.1	21.9	22.8	23.6	24.5	25.3	26.1	27.0	27.8	28.7	29.5	30.4	31.2	32.0	32.9	33.7	34.6	35.4	36.3	37.1	37.9	38.8	
1.56	18.9	19.7	20.5	21.4	22.2	23.0	23.8	24.7	25.5	26.3	27.1	27.9	28.8	29.6	30.4	31.2	32.1	32.9	33.7	34.5	35.3	36.2	37.0	37.8	38.6
1.58	18.4	19.2	20.0	20.8	21.6	22.4	23.2	24.0	24.8	25.6	26.4	27.2	28.0	28.8	29.6	30.4	31.2	32.0	32.8	33.6	34.4	35.3	36.1	36.9	37.7
1.60	18.0	18.8	19.5	20.3	21.1	21.9	22.7	23.4	24.2	25.0	25.8	26.6	27.3	28.1	28.9	29.7	30.5	31.3	32.0	32.8	33.6	34.4	35.2	35.9	36.7
1.62		18.3	19.1	19.8	20.6	21.3	22.1	22.9	23.6	24.4	25.1	25.9	26.7	27.4	28.2	29.0	29.7	30.5	31.2	32.0	32.8	33.5	34.3	35.1	35.8
1.64		17.8	18.6	19.3	20.1	20.8	21.6	22.3	23.1	23.8	24.5	25.3	26.0	26.8	27.5	28.3	29.0	29.7	30.5	31.2	32.0	32.7	33.5	34.2	34.9
1.66			18.1	18.9	19.6	20.3	21.0	21.8	22.5	23.2	24.0	24.7	25.4	26.1	26.9	27.6	28.3	29.0	29.8	30.5	31.2	31.9	32.7	33.4	34.1
1.68			17.7	18.4	19.1	19.8	20.5	21.3	22.0	22.7	23.4	24.1	24.8	25.5	26.2	26.9	27.6	28.3	29.1	29.8	30.5	31.2	31.9	32.6	33.3
1.70				18.0	18.7	19.4	20.1	20.8	21.5	22.1	22.8	23.5	24.2	24.9	25.6	26.3	27.0	27.7	28.4	29.1	29.8	30.4	31.1	31.8	32.5
1.72				17.6	18.3	18.9	19.6	20.3	21.0	21.6	22.3	23.0	23.7	24.3	25.0	25.7	26.4	27.0	27.7	28.4	29.1	29.7	30.4	31.1	31.8
1.74					17.8	18.5	19.2	19.8	20.5	21.1	21.8	22.5	23.1	23.8	24.4	25.1	25.8	26.4	27.1	27.7	28.4	29.1	29.7	30.4	31.0
1.76						18.1	18.7	19.4	20.0	20.7	21.3	22.0	22.6	23.2	23.9	24.5	25.2	25.8	26.5	27.1	27.8	28.4	29.1	29.7	30.3
1.78						17.7	18.3	18.9	19.6	20.2	20.8	21.5	22.1	22.7	23.4	24.0	24.6	25.2	25.9	26.5	27.1	27.8	28.4	29.0	29.7
1.80							17.9	18.5	19.1	19.8	20.4	21.0	21.6	22.2	22.8	23.5	24.1	24.7	25.3	25.9	26.5	27.2	27.8	28.4	29.0
1.82							17.5	18.1	18.7	19.3	19.9	20.5	21.1	21.7	22.3	22.9	23.5	24.2	24.8	25.4	26.0	26.6	27.2	27.8	28.4
1.84								17.7	18.3	18.9	19.5	20.1	20.7	21.3	21.9	22.4	23.0	23.6	24.2	24.8	25.4	26.0	26.6	27.2	27.8
1.86									17.9	18.5	19.1	19.7	20.2	20.8	21.4	22.0	22.5	23.1	23.7	24.3	24.9	25.4	26.0	26.6	27.2
1.88										18.1	18.7	19.2	19.8	20.4	20.9	21.5	22.1	22.6	23.2	23.8	24.3	24.9	25.5	26.0	26.6
1.90											18.3	18.8	19.4	19.9	20.5	21.1	21.6	22.2	22.7	23.3	23.8	24.4	24.9	25.5	26.0
1.92												18.4	19.0	19.5	20.1	20.6	21.2	21.7	22.2	22.8	23.3	23.9	24.4	25.0	25.5
1.94													18.6	19.1	19.7	20.2	20.7	21.3	21.8	22.3	22.9	23.4	23.9	24.4	25.0

Continues next page

Determination of Body Mass Index (continued from previous page)

height (m) \ weight (kg)	92	94	96	98	100	102	104	106	108	110	112	114	116	118	120	122	124	126	128	130	132
1.44																					
1.46																					
1.48																					
1.50																					
1.52																					
1.54	38.8	39.6																			
1.56	37.8	38.6	39.4																		
1.58	36.9	37.7	38.5	39.3																	
1.60	35.9	36.7	37.5	38.3	39.1																
1.62	35.1	35.8	36.6	37.3	38.1	38.9															
1.64	34.2	34.9	35.7	36.4	37.2	37.9	38.7	39.4													
1.66	33.4	34.1	34.8	35.6	36.3	37.0	37.7	38.5	39.2												
1.68	32.6	33.3	34.0	34.7	35.4	36.1	36.8	37.6	38.3	39.0	39.7										
1.70	31.8	32.5	33.2	33.9	34.6	35.3	36.0	36.7	37.4	38.1	38.8										
1.72	31.1	31.8	32.4	33.1	33.8	34.5	35.2	35.8	36.5	37.2	37.9	38.5	39.2								
1.74	30.4	31.0	31.7	32.4	33.0	33.7	34.4	35.0	35.7	36.3	37.0	37.7	38.3								
1.76	29.7	30.3	31.0	31.6	32.3	32.9	33.6	34.2	34.9	35.5	36.2	36.8	37.4	38.1							
1.78	29.0	29.7	30.3	30.9	31.6	32.2	32.8	33.5	34.1	34.7	35.3	36.0	36.6	37.2	37.9						
1.80	28.4	29.0	29.6	30.2	30.9	31.5	32.1	32.7	33.3	34.0	34.6	35.2	35.8	36.4	37.0	37.7					
1.82	27.8	28.4	29.0	29.6	30.2	30.8	31.4	32.0	32.6	33.2	33.8	34.4	35.0	35.6	36.2	36.8	37.4				
1.84	27.2	27.8	28.4	28.9	29.5	30.1	30.7	31.3	31.9	32.5	33.1	33.7	34.3	34.9	35.4	36.0	36.6	37.2			
1.86	26.6	27.2	27.7	28.3	28.9	29.5	30.1	30.6	31.2	31.8	32.4	33.0	33.5	34.1	34.7	35.3	35.8	36.4	37.0	37.6	
1.88	26.0	26.6	27.2	27.7	28.3	28.9	29.4	30.0	30.6	31.1	31.7	32.3	32.8	33.4	34.0	34.5	35.1	35.6	36.2	36.8	37.3
1.90	25.5	26.0	26.6	27.1	27.7	28.3	28.8	29.4	29.9	30.5	31.0	31.6	32.1	32.7	33.2	33.8	34.3	34.9	35.5	36.0	36.6
1.92	25.0	25.5	26.0	26.6	27.1	27.7	28.2	28.8	29.3	29.8	30.4	30.9	31.5	32.0	32.6	33.1	33.6	34.2	34.7	35.3	35.8
1.94	24.4	25.0	25.5	26.0	26.6	27.1	27.6	28.2	28.7	29.2	29.8	30.3	30.8	31.4	31.9	32.4	32.9	33.5	34.0	34.5	35.1
1.96	23.9	24.5	25.0	25.5	26.0	26.6	27.1	27.6	28.1	28.6	29.2	29.7	30.2	30.7	31.2	31.8	32.3	32.8	33.3	33.8	34.4
